# Assessment of cardiac biomarker “point-of-care” testing as postmortem diagnostic tool

**DOI:** 10.1007/s00414-025-03517-y

**Published:** 2025-05-29

**Authors:** Jan Michael Federspiel, Mattias Kettner, Stefan Potente, Sara Heinbuch, Constantin Lux, Marcel A. Verhoff, Frank Ramsthaler

**Affiliations:** 1 Institute for Legal Medicine, Faculty of Medicine, Saarland University, Campus Homburg, Building 49.1, Kirrberger Straße 100, 66421 Homburg, Saarland, Germany; 2 Institute of Legal Medicine, University Hospital of Frankfurt, Goethe University, Kennedyallee 104, 60596 Frankfurt (Main), Hessen, Germany; 3 Department of Forensic Medicine and Imaging, Institute of Forensic Medicine, University of Zurich, Winterthurerstrasse 190/52, 8057 Zurich, Switzerland

**Keywords:** Cardiac biomarkers, Point-of-care testing, Postmortem blood analysis, Cardiac death, Heart failure, Diagnostic performance

## Abstract

In cardiac death, some entities, such as arrhythmia or nonocclusive myocardial ischemia, are not associated with clear and certain macroscopic surrogates of cardiac death. Cardiac biomarker point-of-care testing (POCT) seems suitable for further improving postmortem diagnostics in legal medicine casework. Considering the preanalytic phase, the present study aims to define criteria for blood samples suitable for POCT and assess the diagnostic performance of postmortem cardiac biomarker POCT. A fluorescent immunoassay device was used. The biomarkers assessed were myoglobin, brain-type natriuretic peptide (BNP), N-terminal proBNP, creatine kinase muscle-brain type, and cardiac troponin I. Blood was obtained from the intrapericardial inferior vena cava. In a prestudy, criteria for the selection of blood samples were established and the biomarker stability over time, test reliability and reproducibility of postmortem cardiac biomarker analyses were assessed. Afterward, blood samples from 150 autopsied individuals were evaluated for their diagnostic performance and compared with findings from autopsy as the postmortem diagnostic gold standard. In doing so, the assessed biomarkers provided valid and reproducible results. Cardiac troponin I yielded the highest sensitivity for detecting cardiac death, whereas BNP had the highest specificity and positive predictive value for detecting cardiac death. Markers of myocardial damage had better negative than positive predictive value. NT-proBNP and BNP POCT seem applicable to support diagnosis of death associated with congestive heart failure. Postmortem cardiac biomarker POCT results need to be interpreted in conjunction with all available information, i.e., autopsy findings, medical history, investigatory results, and other test results.

**Supplementary Information:**

The online version contains supplementary material available at 10.1007/s00414-025-03517-y.

## Introduction

Cardiovascular diseases are the leading cause of death worldwide [1]. These diseases are frequently encountered in routine legal medical casework. In some of these entities, such as nonocclusive myocardial ischemia, macroscopic findings are lacking [2]. Additionally, established definitions of several cardiovascular diseases, for example, congestive heart failure (HF; all abbreviations are summarized in Supplemental File–AppendixA) are based on clinical observations. Major guidelines define HF as a syndrome with different signs (e.g., edema) and symptoms, such as dyspnea, that can be associated with elevated cardiac biomarker (cBM) levels [3]. However, such symptoms are not assessable during an autopsy. Therefore, the definition requires adaptation to the postmortem setting, and subsequent analyses following autopsy can be necessary to evaluate the functional state of the cardiovascular system around the time of death. A subsequent analysis that could help determine the actual cause of death could be postmortem measurement of cBM levels [4].

cBM levels are usually obtained through laboratory or extralaboratory analysis of routine blood collection. The latter is also termed point-of-care testing (POCT) [5, 6]. POCT quickly and simply yields test results with rather modest equipment requirements [5], which is usually necessary in legal medicine casework. In the clinical setting, cBM POCT is a well-established [7, 8], proven method for obtaining reliable test results [8, 9] that strongly agree with laboratory test results [10] despite their lower accuracy than laboratory tests [11]. POCT has already been established in the postmortem setting for screening for illegal substances [12] or postmortem diagnosis of sepsis [13]. Thus, cBM POCT appears to be ideal for use in legal medicine as a *“deadside”* [14] diagnostic tool to swiftly and cost-efficiently complement the autopsy and thus postmortem diagnosis of cardiac deaths.

The value of cBM measurement in a postmortem setting has been analyzed in many studies (Table 1), but the results have been contradictory. Some studies have shown that cBM analysis can improve the ability to identify cardiac deaths [15], whereas others have revealed inconsistent results and conclusions, especially regarding postmortem stability [16, 17]. Additionally, the sampling [18–21] or the combination of different cBMs [22] has been scrutinized. Such skepticism seems reasonable given that the preanalytical phase is the most frequent period in which analytical problems occur [6, 23] and the value of postmortem cBM testing in this phase has not been studied (systematic literature search Supplemental File–Appendix B). Additionally, these issues are not reported in the available meta-analyses and systematic reviews [4, 24–26].

**Table 1:** Previous studies on the value of postmortem cBM testing by publication year

	Reference/Year	Case number	Analyzed body fluid	Analyzed marker(s)	Longest PMI
1	[27]/1988	28	Serum	CK-MB, cTnI	40 h
2	[28]/2004	102	Serum	cTnT	75 h
3	[29]/2006	405	Serum, pericardial fluid	cTnT	48 h
4	[30]/2006	171	Serum; Pericardial fluid	cTnT	48 h
5	[31]/2008	96	Femoral blood, serum, vitreous humor, pericardial fluid	NT-proBNP	≤ 24 h
6	[32]/2008	53	Serum	cTnI	61 h
7	[33]/2009	257	Serum, cerebrospinal fluid	cTnI, CK-MB	48 h
8	[34]/2013	230	Serum, pericardial fluid	cTnT	141 h
9	[35]/2016	58	Serum, pericardial fluid	hs cTnT	34 h
10	[36]/2017	20	EDTA anticoagulated blood	CK-MB, LDH, cTnI, cTnT	8 h
11	[37]/2018	140	Serum	cTnT	< 24 h

Summary of previous studies on the value of postmortem testing of cardiac biomarkers not otherwise discussed or mentioned. *Abbreviations: BNP* brain-type natriuretic peptide, *CK-MB* creatine kinase – muscle-brain-type, *cTnI* cardiac troponin I, *(hs) cTnT* (high sensitivity) cardiac troponin T, *EDTA *ethylenediaminetetraacetic acid, *LDH* lactate dehydrogenase, *NT-proBNP* N-terminal pro-brain-type natriuretic peptide, *PMI* postmortem interval

**Fig. 1:**
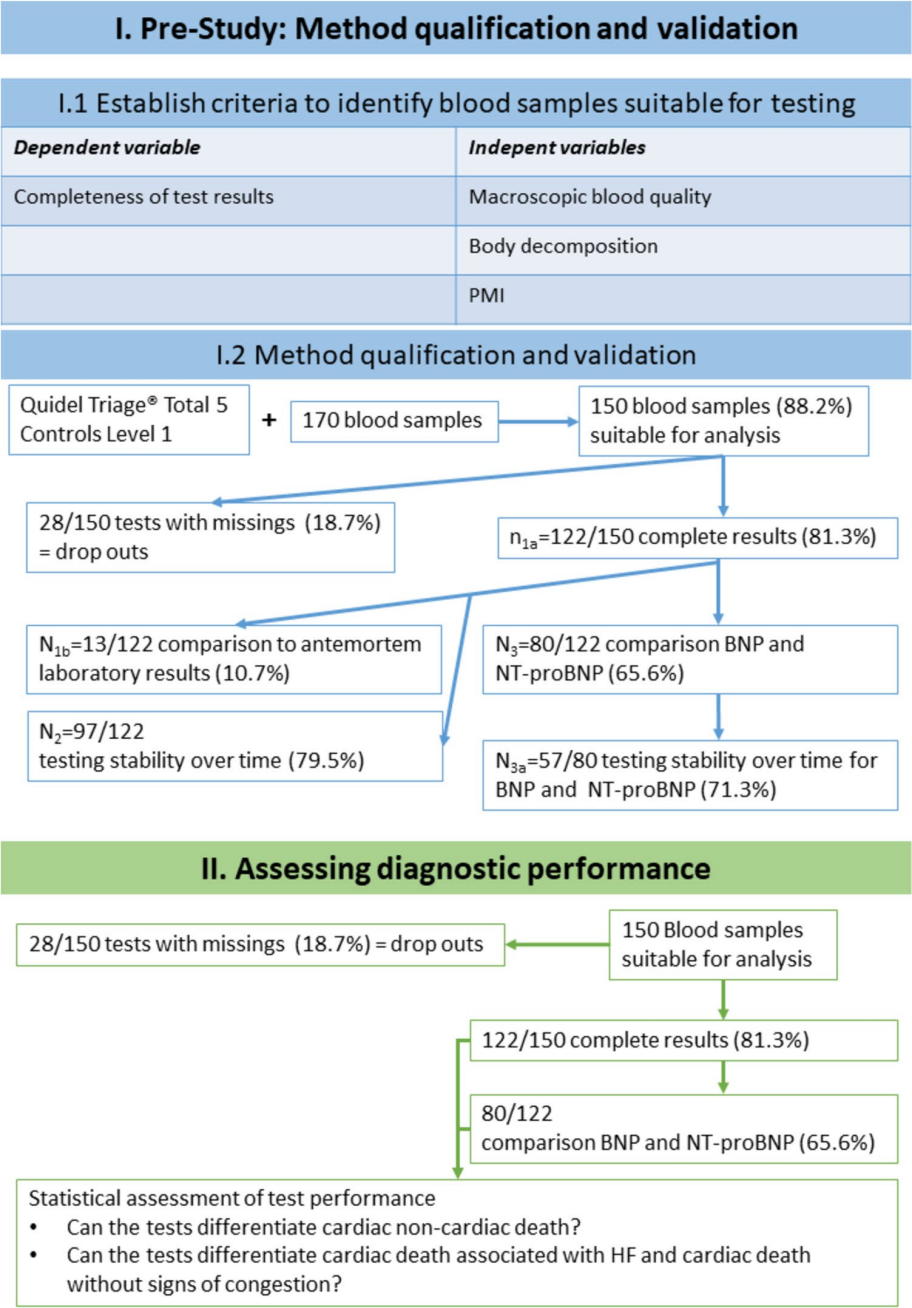
Study flow chart. The prestudy is marked in blue. The main study is labeled green. Details regarding the criteria for sample selection can be found in Table 2. *Abbreviations*: BNP – brain-type natriuretic peptide; HF – heart failure; NT-proBNP – N-terminal pro-brain-type natriuretic peptide. PMI – postmortem interval

In the present study, the following five POCT cBMs were assessed: creatine kinase muscle-brain-type (CK-MB), cardiac troponin I (cTnI), myoglobin (myo), brain-type natriuretic peptide (BNP), and N-terminal proBNP (NT-proBNP). This panel was chosen because of its outstanding importance in identifying ischemic heart disease (the leading cause of death worldwide [38]) and HF (a global health burden with high mortality affecting approximately 64 million people worldwide [39]). For the present study, a stepwise approach was applied (the study workflow is summarized in Fig. 1). First, a prestudy assessing the preanalytical phase was conducted to define criteria for the selection of blood samples suitable for postmortem cBM POCT, assess postmortem POCT result reproducibility and reliability, and compare the postmortem POCT results with available clinical laboratory test results acquired shortly before death. Second, the diagnostic performance of postmortem cBM POCT was assessed. To do so, we analyzed whether the assessed cBMs (single or combined) supported the diagnosis of cause of death and helped to differentiate between cardiac and noncardiac deaths. Additionally, we analyzed whether BNP and NT-pro BNP differ in the postmortem setting in terms of their diagnostic value.

## Materials and methods

The current “gold standard” for determining the cause of death is autopsy [40]. Thus, to determine the value of postmortem cBM POCT, whether this POCT can complement autopsy by providing additional information or validate the autoptic diagnosis was assessed. The workflow is summarized in Fig. 1. The present study was conducted with the consent of the local ethics committee (vote number: 277/2022).

### Analyzer device and quality control

The AlereTM Triage® MeterPro was used as the analyzer device (a portable fluorescence immunoassay instrument; in the meantime, Alere™ was acquired by Quidel®). According to the manufacturer’s instructions, Quidel® quality control samples were used for quality control, and the analyzer was repeatedly calibrated during the study period. For the present study, the Alere™ Triage® Profiler SOB™ (Shortness of Breath) and the Alere™ Triage® NT-proBNP test kits were used. These kits are based on murine monoclonal antibodies directed against the examined markers. Approximately 50 µl of blood is required for each test with one of these kits. A limiting factor of these kits is that they do not provide a measurement of so-called high-sensitivity cTn, which is recommended by major guidelines [41].

### Analyzed cBMs and threshold values according to the manufacturer

Because of postmortem alterations in blood samples [42], a combination of five different biomarkers was chosen: (a) cTnI was used as a marker for myocardial damage, such as myocardial ischemia or thoracic trauma [41] (threshold values: 0–0.4 ng/ml); (b) myo was used as a general marker of striated muscle damage, for example, damage caused by trauma [43] (threshold values: 0–107 ng/ml); (c) CK-MB was used as an isoenzyme that is more specific for damage to the myocardium [44] (threshold values: 0–4.3 ng/ml); and (d) BNP (threshold values: 0–100 pg/ml) and (e) NT-proBNP (threshold values: 0–125 (300) pg/ml) were used as markers of increased cardiac wall tension [45] and are thus relevant for the diagnosis of HF [3].

### Analyzed samples

All blood samples analyzed were collected during complete medico-legal autopsies. Owing to the circumstances of the severe acute respiratory syndrome coronavirus 2 (SARS-CoV-2) pandemic, the analyses took two years. Blood was obtained from the intrapericardial inferior vena cava at an early stage of the autopsy and anticoagulated with ethylenediaminetetraacetic acid (EDTA) according to the Alere™ instructions to facilitate full blood analyses (Sarstedt S-Monovette®; Sarstedt, Germany, 2.7 ml K3E – 1.6 mg EDTA/ml).

### Prestudy on criteria for blood sample suitability for analysis and reproducibility

Owing to known postmortem artifacts associated with cell death and putrefaction, the focus of this part of the study was on establishing criteria for the selection of blood samples to allow reasonable test results. Additionally, biomarker stability in blood samples and test reliability were assessed. To do so, the completeness of test results was seen as a variable dependent on macroscopic blood quality, the corpse’s putrefaction grade, and the postmortem interval (PMI) (i.e., independent variables). Each independent variable was subdivided into an ordinal scale (Table 2). To assess the criteria for the selection of blood samples suitable for POCT, the POCT results in the different ordinal categories for PMI, macroscopic blood quality, and putrefaction of the corpse (Table 2) were compared. To assess the reliability of the postmortem cBM POCT results, quadruple testing was performed when possible (i.e., suitable for the institute’s routine). The first two tests were performed within 30 min after autopsy (tests T_1a_ and T_1b_). Twenty-four hours later, the double test was repeated within 30 min (tests T_2a_ and T_2b_). The pre-study is summarized in Fig. 1.

**Table 2:** Grouping to assess criteria for blood samples suitable for analysis

Postmortem interval [h]	Macroscopic blood quality	Putrefaction	Completeness of test results per case
**A** ≤ 24	**A + + + **exclusively thin fluid, no fat globules	**0** no macroscopically discernible signs of degradation, rigor mortis present	**R + –**Results of all analyzed parameters were available
**B** > 24 and ≤ 48	**A + + **fluid, no fat globules	**I** spot-like greenish discolorations of the lower abdomen, rigor mortis present	**R(+)** – Some analyzed parameters showed error messages from the analyzer
**C** > 48 and ≤ 72	**A + **viscous, no fat globules	**II** extensive discolorations of the skin due to putrefaction including marbling, skin slippage, and decomposition fluid draining from at least one orifice, rigor mortis resolved	**R(-)** – All parameters showed an error message from the analyzer
**D** > 72 and ≤ 96	**A** fluid, fat globules	**III** any putrefaction exceeding grade II	
**E** > 96	**B** fluid, sporadic clots		
	**C** viscous, fat globules, clots		

The table provides the definitions of the different groups applied for the identification of blood samples suitable for point-of-care testing

#### Prestudy statistical analysis

MedCalc Version 19.1 (MedCalc Software Ltd., Ostend, Belgium) was used for the statistical analysis. A significance level of α = 0.05 was assumed; thus, p values < 0.05 were considered statistically significant. To compare the first and second measurements (T_1a_ vs. T_1b_ and T_2a_ vs. T_2b_), Bland‒Altman plots [46, 47] were employed. The second measurements were plotted against the respective first measurements, which were defined as the so-called “gold standard” for this analysis [48]. Additionally, the coefficients of variation (CVs) of the early double measurements (T_1a_ and T_1b_) were calculated to determine reproducibility. Thus, the CVs were used to estimate the within-run imprecision directly as expounded by the MedCalc information function according to Jones and Payne 1997 [49]. Additionally, mountain plots were created by computing a percentile for each ranked difference between a second measurement and a reference first measurement [48, 50]. These plots are complementary to the Bland‒Altman plots only because a nonnormal distribution was observed.

### Main study on the diagnostic performance of cBM POCT’s

The diagnostic performance of cBM POCT was evaluated using blood samples suitable for testing according to the criteria defined in the prestudy. During the main study, the duration of the preanalytic phase was considered by performing two tests within 30 min after the autopsy (T_1a_ and T_1b_). A summary of the main study is provided in Fig. 1. All cases allowing the performance of postmortem POCT (e.g., no time constraints in a pressing case), without advanced putrefaction (e.g., partial skeletonization), and a patient age of at least 18 years were included.

#### Grouping by cause of death

Grouping of the analyzed cases was necessary to facilitate statistical analysis of the diagnostic performance. To allow grouping, it was necessary to define in which cases autopsy findings were evidence for cardiac death. With (NT-pro)BNP being an indicator of increased wall tension [51], a definition of autoptic evidence of death associated with congestive HF was also needed. The definition applied is an extension of an already established postmortem HF definition by Sabatasso et al. [52]:

**Group 0 – Noncardiac death group:** This group served as the reference group. Cases were assigned to this group if clinical and/or autopsy findings suggested any noncardiac death (e.g., trauma).

**Group 1 – Cardiac death in general:** Cases were assigned to this group if clinical and/or autopsy findings strongly suggested a cardiac cause of death. To assess the diagnostic performance of BNP and NT-proBNP, cardiac death not associated with congestive HF (Group 1a) and cardiac death associated with signs of congestive HF (Group 1b) were differentiated.

**Group 1a – Cardiac death not associated with congestive HF:** Cases were assigned to this group in the absence of later defined signs of or conditions associated with chronic congestion. Major indicators for assignment to Group 1a were (1) nontraumatic pericardial tamponade; (2) extensive acute myocardial infarction; (3) fresh acute coronary occlusion without a demarked infarction area; (4) severe coronary multivessel disease with the presence of multiple myocardial scarred areas without signs of chronic congestion; (5) histologically confirmed severe myocardial fibrosis and/or lipomatosis affecting > 50% of the analyzed tissue slides (grading adapted from [53]) and other evidence for arrhythmogenicity without signs of chronic congestion; (6) marked ventricular thickening (left ventricular posterior wall ≥ 15 mm; right ventricular outflow tract ≥ 5 mm) resulting in a heart weight ≥ 500 g; (7) histological evidence of myocarditis; (8) macroscopic evidence of acute aortic or mitral valve regurgitation, such as aortic valve commissural teardown in Stanford Type A aortic dissection; (9) acute pulmonary embolism with significant central occlusion of the pulmonary arteries; and (10) clinical data supporting cardiac death not belonging to Group 1b (e.g., evidence of ventricular fibrillation due to increased blood levels of potassium).

**Group 1b – Cardiac death associated with signs of congestive HF:** Cases were assigned to this group in the absence of competing causes of death and the observation of at least one of the following conditions indicating chronic congestion: (1) contemporaneous presence of at least two macroscopic signs of congestion (i.e., edema and/or filled jugular veins and/or anasarca); (2) macroscopic signs of chronic congestion (i.e., nutmeg liver and/or fibrous thickening of the splenic capsule and/or dilated atria); (3) macroscopic signs of chronic heart disease (i.e., cardiac enlargement with marked rounding of the apex) associated with hints for chronic congestion; (4) macroscopic signs of acute chronic congestion (i.e., markedly increased blood content in the liver, lungs, and kidneys resulting in increased weights of chronically congested organs); (5) macroscopic signs of prolonged ventricular stasis (i.e., organized thrombus in the left ventricle); and (6) clinical parameters supporting cardiac death associated with congestion (e.g., reports of intensive care unit stay with chronically elevated right atrial pressures).

The assignment of each case to one of the groups was based on all the information available at the end of the autopsy (i.e., autopsy findings, excerpts of the individual’s medical history, other investigatory results, etc.).

#### Main study statistical analysis

MedCalc Version 19.1 (MedCalc Software Ltd., Ostend, Belgium) was again used (significance level α = 0.05). To assess the diagnostic performance of postmortem cBM POCT, receiver operating characteristic (ROC) curve analyses were performed [54, 55]. Each cBM was evaluated for its ability to differentiate cardiac and noncardiac deaths. The underlying assumption for the ROC curve analysis was that an area under the ROC curve (AUC) of at least 0.75 indicates a significant difference from the null hypothesis value of 0.5. To estimate the number of cases needed for comparing the AUC values, a sample size calculation was carried out according to Lu et al. [56] using MedCalc’s sample size calculation tool [57]. For that, β = 0.1 was assumed. To compute the estimated sample size, the MedCalc tool requires the incidence of cardiac disease in the assessed cohort (i.e., approximately 27%). The estimation revealed that at least 17 cases of cardiac death associated with congestive HF (group 1b) and at least 63 cases of noncardiac death (i.e., group 0) were needed. Thus, at least 80 cases were necessary in total for the present study.

Additionally, for each marker, ROC curve analysis was carried out for the different test time points (i.e., T_1_ vs. T_2_). The AUC values were also used to compare the diagnostic performance of different cBM combinations [58, 59]. For this purpose, the predicted values for the combined biomarkers were calculated via a logistic regression model (forward and stepwise logistic regression). The applied algorithm works according to the following schemes: (A) cardiac death = log(a + b*marker 1 + c*marker 2…) or (B) cardiac death associated with congestive HF = log (d + e*marker 1 + f*marker 2…). The software output provided the final best-fitting model while considering the prevalence of cardiac death in general or cardiac death associated with congestive HF. The positive predictive value (PPV) and negative predictive value (NPV) were subsequently calculated. The optimal cutoffs were subsequently determined. To further describe the diagnostic performance of the cBM, the sensitivity, specificity, positive likelihood ratio (LH +), and negative likelihood ratio (LH-) were calculated. For the present study, the calculated measures translate to the following meanings: The sensitivity was the likelihood that a test result would be lower than the optimal criterion value if cardiac death in general or cardiac death associated with congestive HF were ruled out. The specificity was regarded as the likelihood that a test result would be positive in the case of a proven cardiac death in general or a proven cardiac death associated with congestive HF. The LH + was the ratio between the likelihood of an elevated test result given the presence of cardiac death in general or cardiac death associated with congestive HF and the likelihood of an increased biomarker level given the absence of cardiac death. The LH- was the ratio of the likelihood of a test result within the reference range in the presence of cardiac death to the likelihood of a normal test result in the absence of cardiac death. The PPV was the likelihood that cardiac death was evident if the biomarker level was above the optimal criterion value. The NPV was the likelihood that cardiac death could not be confirmed if the biomarker level was lower than the cutoff value for all five cBMs.

To assess age as a potential mediator of increased NT-proBNP and BNP levels [60, 61], the nonlinear regression tool in MedCalc was used. This tool automatically chooses a suitable nonlinear regression model on the basis of the analyzed data.

## Results

### Prestudy on criteria for blood sample selection, and method qualification

#### Assessment of criteria for the selection of blood samples

 The missing values according to the assessment criteria applied are shown in Table 3. In summary, complete POCT results were predominantly observed if the PMI was ≤ 96 h (i.e., PMI groups A to D), blood showed no fat globules or clots (i.e., macroscopic blood quality class A +, A + +, or A + + +), and a putrefaction grade ≤ II (explanations of the grades, classes and groups are presented in Table 2).

**Table 3:** Completeness of postmortem POCT results

	PMI (h)					Putrefaction grade				Macroscopic blood quality					
	*A*	*B*	*C*	*D*	*E*	*0*	*I*	*II*	*III*	*A* + + +	*A* + +	*A* +	*A*	*B*	*C*
*R* +	6	14	12	6	0	30	7	1	0	30	6	2	0	0	0
*R(* +*)*	0	1	1	2	1	0	0	1	4	0	0	0	2	1	2
*R(-)*	0	0	0	1	6	0	0	0	7	0	0	0	0	3	4
sum	50					50				50					

Number of cases per applied criterion (Table 2) in which the analyzer partially (“R(+)”) or completely (“R(-)”) failed to analyze the sample. Complete test results are indicated by “R + ”. *Abbreviations*: POCT – point-of-care testing, PMI – postmortem interval

### Reliability, accuracy, and reproducibility of postmortem cBM POCT

**Fig. 2:**
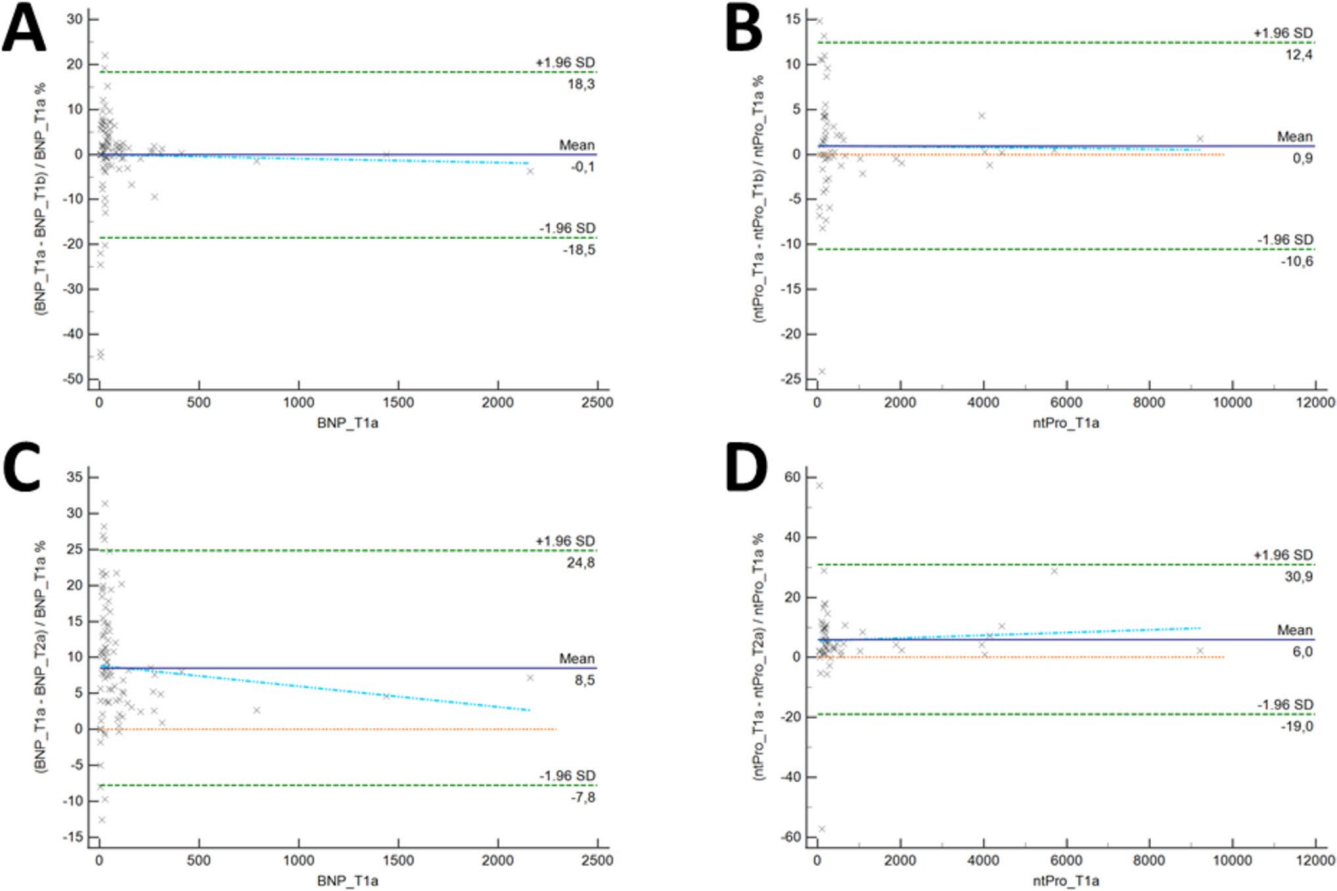
Bland–Altmann plots for double measurements. The upper row shows Bland‒Altmann plots illustrating differences between two measurements shortly after autopsy for BNP (**A**) and NT-proBNP (**B**). The lower row shows Bland‒Altman plots showing the differences between the two measurements 24 h after the autopsy for BNP (**C**) and NT-proBNP (**D**). Differences in values are expressed as percentages of the observations represented on the X-axis. The level of agreement (LoA) is defined as the mean (horizontal purple line) ± 1.96 standard deviation (SD, dotted green lines). The dotted blue lines show the regression of the differences plotted against the reference value. These regressions did not show significant trends of proportional differences in either analysis (green dotted line, p > 0.05). The dotted orange lines indicate the mean difference of the measurements. *Abbreviations*: BNP – brain-type natriuretic peptide; LoA – limit of agreement; NT-proBNP – N-terminal pro-brain-type natriuretic peptide; SD – standard deviation

Double measurements of BNP levels following sampling yielded largely stable values, with a mean difference of −0.1% (95% confidence interval (CI) [18.3% to −18.5%]; Fig. 2A), whereas double measurements after 24 h showed a mean difference of 8.5% (CI [24.8% to −7.8%]; Fig. 2C). With respect to NT-proBNP, the initial double measurements showed a mean difference of 0.9%. (CI [12.4% to −10.6]; Fig. 2B), whereas the double measurement after 24 h yielded a mean difference of 6.0% (CI [30.9% to −19%; Fig. 2D). Regarding cTnI, the early double measurement showed small differences (−0.78%; CI [−2.1295 to 0.5618]), whereas marked differences in the double measurement 24 h after the autopsy were observed (5.54%; CI [1.092510.0054]). In terms of CK-MB, both early (0.29%; CI [−3.29463.8752]) and late (0.15%; CI [−5.49795.8123]) double measurements showed small differences. The largest differences were found in the myo double measurement 24 h after the autopsy (−5.05%; CI [−8.1600 to −1.9530]; early double measurement: −1.31%; CI [−3.0817 to 0.4432]).

In terms of the CVs of the early measurements, the lowest variance was found for NT-proBNP (CV = 2.85%), whereas the greatest variance was observed for cTnI (CV = 12.75%). Mid-level variances were observed for the other markers (myo: CV = 4.15%; BNP: CV = 6.57%; CK-MB: CV = 7.48%). No significant differences in the POCT results of initial duplicate runs were found.

**Fig. 3:**
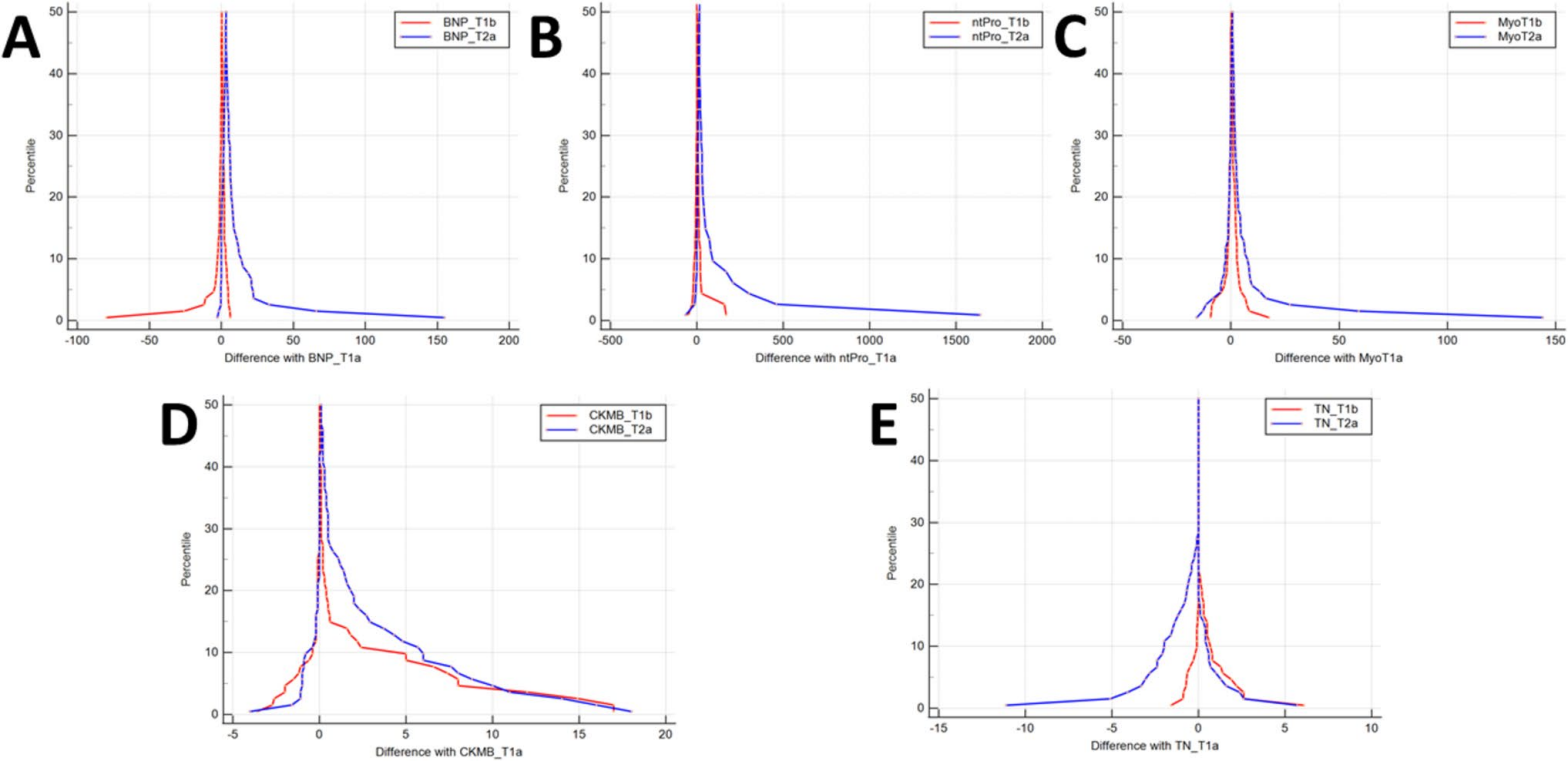
Mountain plots for comparisons of early and late measurements. Mountain plots of double measurements of (**A**) BNP, (**B**) NT-proBNP, (**C**) myo, (**D**) CK-MB, and (**E**) cTnI showing differences of T_1a_ compared to T_1b_ (blue graph) and T_2b_ (red graph). *Abbreviations*: NP – brain-type natriuretic peptide; CK-MB – creatine kinase muscle-brain-type; cTnI – cardiac troponin I; myo – myoglobin; NT-proBNP – N-terminal brain-type natriuretic peptide

For all five markers, the mountain plots comparing T_1a_ to T_1b_ and T_2a_ (Fig. 3) were centered at approximately 0 (i.e., small differences between the measurements). However, for all assessed biomarkers, a shallower slope for the comparison with T_2a_ indicated greater differences between the different POCT results.

In those cases with available antemortem laboratory results (n = 13), the NT-proBNP level showed an average decrease to 84.9% (CI for the arithmetic mean [71% to 98.8%]) of the clinically determined level. Compared with the antemortem laboratory results, no postmortem increase in the NT-proBNP level was observed. For cTnI, the differences in ante- and postmortem cTnI levels strongly depended on the PMI. In nine patients, the postmortem cTnI level was lower than the antemortem clinical test result (all patients had either a PMI ≤ 36 h or, interestingly, ≥ 48 h). In four instances, a postmortem increase in cTnI levels compared with the antemortem clinical test results was observed (all cases with a PMI ≥ 36 h).

### Main study on the diagnostic performance of cBM POCT

In the ROC curve analysis, a diagnostic benefit for BNP and NT-proBNP was observed in cases of cardiac death associated with congestive HF (Group 1b; Table 4). When the AUC values of BNP and NT-proBNP were compared, no statistically significant differences were observed for the diagnoses of cardiac death associated with congestive HF (p = 0.68) and cardiac death in general (Group 1, p = 0.15). A stepwise logistic regression revealed that for a dichotomous decision on the diagnosis of cardiac death (i.e., “yes or no”), a combination of BNP and cTnI was valuable (correct decision “yes” in 58.14%; correct decision “no” in 86.08%; overall, 76.23% of the cases correctly classified). In the aforementioned logistic regression, good results for the diagnosis of cardiac death associated with congestive HF were shown for BNP alone (correct decision “yes” in 51.72%; correct decision “no” in 97.8%; overall 86.67% of the cases correctly classified).

**Table 4:** Comparative ROC curve analysis of BNP and NT-proBNP

Variable	AUC	SE	95%-CI	p
*ROC curve analysis of BNP and NT-proBNP – Acute on chronic congestion (Group 1b)*				
*BNP_T1a*	0.896	0.041	0.82 to 0.94	< 0.0001
*BNP_T2a*	0.893	0.048	0.81 to 0.94	< 0.0001
*NT-proBNP_T1a*	0.917	0.043	0.83 to 0.96	< 0.0001
*NT-proBNP_T2a*	0.887	0.056	0.77 to 0.95	< 0.0001
*ROC curve analysis of BNP and NT-proBNP – Cardiac death (Group 1)*				
*BNP_T1a*	0.661	0.062	0.57 to 0.74	< 0.009
*BNP_T2a*	0.609	0.072	0.50 to 0.70	< 0.13
*NT-proBNP_T1a*	0.823	0.054	0.72 to 0.89	< 0.0001
*NT-proBNP_T2a*	0.769	0.071	0.63 to 0.87	< 0.001

The different time points T_1a/b_ and T_2a/b_ are explained in the Materials and Methods section. *Abbreviations: AUC* area under the receiver operating characteristic (ROC) curve, *BNP* brain-type natriuretic peptide, *CI* confidence interval, *NT-proBNP* N-terminal brain-type natriuretic peptide, *SE* standard error

In terms of the myocardial damage markers, analysis of the ROC curves for cTnI, CK-MB, and Myo in cases of congestive HF did not reveal statistically significant differences in the AUC values. The values ranged from 0.7 for cTnI to 0.72 for CK-MB. Among the cases of cardiac death in general (Group 1) and noncardiac death (Group 0), cTnI had the highest AUC value (0.8). A statistically significant difference (p = 0.036) was found solely by comparing the AUC values of cTnI and myo. PPVs for the diagnosis of cardiac death increased with increasing levels of cTnI, CK-MB, and myo, but no linear correlation was found. Interestingly, the likelihood of a correct diagnosis due to cTnI levels markedly increased if the levels were far above the clinical threshold, i.e., ≥ 30 ng/ml (Table 5).

**Table 5:** Summary diagnostic performance of postmortem cBM POCT

	Optimal value (cut-off)	Sensitivity	Specificity	LH +	LH-	PPV	NPV
*Overall Cardiac Death (Group 1)*							
*cTnI [ng/ml]*	> 1.1	81.4%	79.7%	4.0	0.2	68.6%	88.7%
*Myo [ng/ml]*	> 33.0	67.4%	75.9%	2.8	0.4	60.4%	81.1%
*CK-MB [ng/ml]*	> 2.6	81.4%	68.3%	2.5	0.2	58.3%	87.1%
*BNP [pg/ml]*	> 81.3	58.1%	89.8%	5.7	0.4	75.8%	79.8%
*NT-proBNP [pg/ml]*	> 209	77.4%	83.6%	4.7	0.2	75.0%	85.4%
*Cardiac death associated with HF (Group 1b)*							
*cTnI [ng/ml]*	> 0.4	72.4%	63.4%	1.9	0.4	38.2%	88.1%
*Myo [ng/ml]*	> 33.0	72.4%	70.9%	2.4	0.3	43.7%	89.3%
*CK-MB [ng/ml]*	> 80.0	24.1%	97.8%	11.1	0.7	77.8%	8.5%
*BNP [pg/ml]*	> 74.7	86.2%	90.3%	8.9	0.1	73.5%	95.5%
*NT-proBNP [pg/ml]*	> 390	78.2%	96.4%	22.3	0.2	90.0%	91.7%

For definitions of the different groups, please see the Materials and Methods section. *Abbreviations: cBM* cardiac biomarker, *BNP* brain-type natriuretic peptide, *CK-MB* creatine kinase muscle-brain-type, *cTnI* cardiac troponin I, *HF* heart failure, *LH + *positive likelihood ratio, *LH- *negative likelihood ratio, *myo* myoglobin, *NPV* negative predictive value, *NT-proBNP* N-terminal brain-type natriuretic peptide, positive predictive value

Overall, a greater diagnostic benefit of postmortem cBM POCT was observed in the detection of cardiac death associated with congestive HF than in cases with cardiac death in general. The significance of NPVs exceeded that of PPVs in all instances. Logistic regression analysis revealed a rather mild increase in diagnostic confidence when different markers were combined, with a classification success rate of approximately 80% (cTnI and BNP). After the criteria for the selection of blood samples suitable for postmortem cBM POCT were applied, an error rate of 22.9% was observed.

**Fig. 4:**
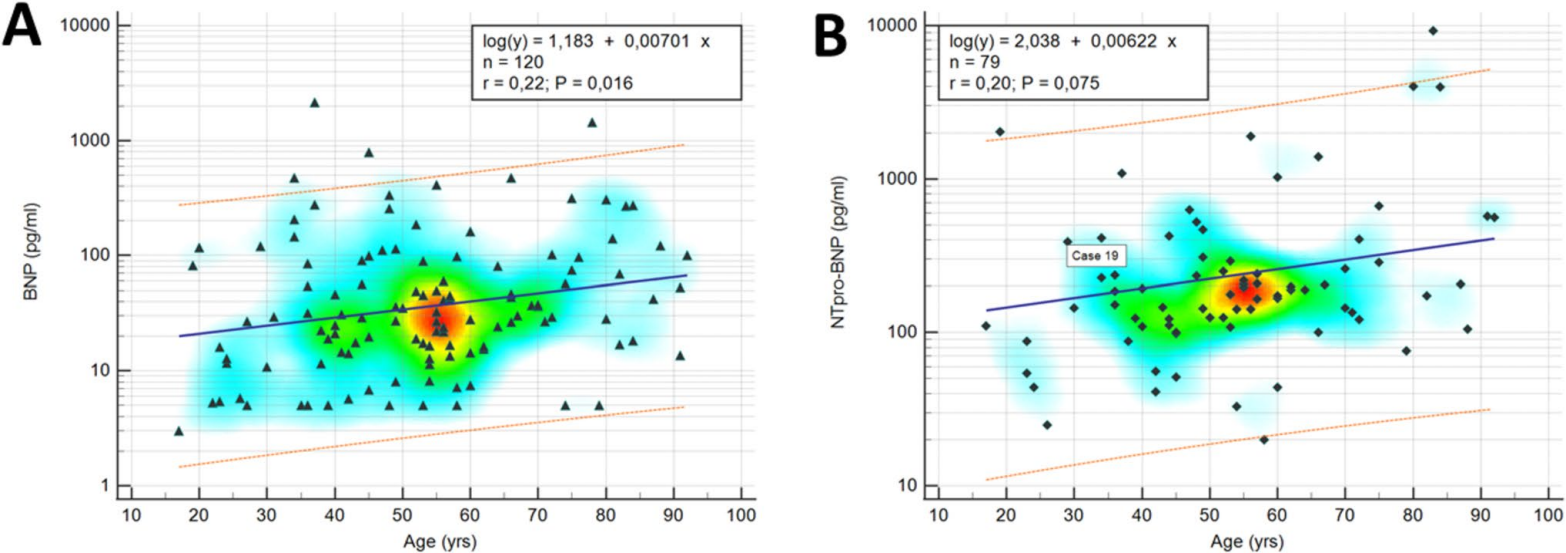
BNP and NT-proBNP regression analysis. Mountain plots of double measurements of (**A**) BNP, (**B**) NT-proBNP, (**C**) myo, (**D**) CK-MB, and (**E**) cTnI showing differences in T_1a_ compared with T_1b_ (blue graph) and T_2b_ (red graph). *Abbreviations:* BNP – brain-type natriuretic peptide; CK-MB – creatine kinase muscle-brain-type; cTnI – cardiac troponin I; myo – myoglobin; NT-proBNP – N-terminal brain-type natriuretic peptide

Clinically, aging has been found to interfere with the blood levels of natriuretic peptides [60, 61]. Similarly, a nonlinear regression analysis of age and postmortem levels of BNP and NT-proBNP revealed a statistically significant positive but weak correlation with age (BNP: r = 0.22; p = 0.016; NT-proBNP: r = 0.20; p = 0.075; Fig. 4).

## Discussion

In general, blood tests are useful and powerful adjuncts to autopsies, allowing otherwise impossible appraisements (e.g., postmortem assessment of blood glucose control during the lifetime in a diabetic individual [62]). Although powerful, such postmortem blood analyses are challenging due to postmortem changes such as the breakdown of active membrane transport processes [42] or putrefaction [63]. Therefore, the applicability and diagnostic performance of postmortem cBM POCT (cTnI, CK-MB, Myo, BNP, and NT-proBNP) was assessed in the present study. The prestudy demonstrated the applicability of postmortem cBM POCT and identified criteria for the selection of blood samples suitable for POCT. The main study, in which the diagnostic performance of postmortem cBM POCT in legal medicine routine casework was assessed, revealed a higher NPV than PPV of the assessed biomarkers.

### Importance of analyses after autopsy

Autopsy is considered the “gold standard” for determining the cause of death [40]. However, the autopsy itself is limited to delineated macroscopic findings [40], such as pericardial tamponade. To determine causes of death associated with limited or no distinct macroscopic findings (e.g., intoxications or arrhythmias), autopsies have been complemented by an ever-increasing pool of supportive histological [64], immunohistological [65], biochemical [66], toxicological [67], microbiological [68], and postmortem imaging [69] methods. Additionally, laboratory tests can be used to identify the cause of death (for example, [13]) and to make postmortem diagnoses of cardiac diseases [70]. Correspondingly, postmortem biochemical evaluation has been recommended for the investigation of sudden cardiac death despite the influence of agonal processes on cBM levels [25].

Along with the effects of agonal processes, additional limitations are faced in postmortem cBM analyses. Therefore, entities without distinct macroscopic findings are usually associated with elevated cBM levels. For example, atrial fibrillation is associated with increased cBM levels [45] but without clear pathological or anatomical substrates [71]. Such diseases can potentially confound postmortem cBM POCT results, as they might cause increased cBM levels despite a rather unremarkable heart, leading to the wrong assumption of postmortem artifacts. Additionally, impaired renal function can lead to increased biomarker levels (e.g., cTn [72] or BNP and NT-proBNP [73]), and many other diseases or circumstances interfere with postmortem biomarker levels (e.g., ventricular arrhythmia increases Tn [74] and BNP levels [75]; trauma can increase Tn levels [76]; sepsis can be associated with elevated Tn [77, 78] and NT-proBNP [78] levels; electrocution elevates cTn levels [79]; asphyxia can increase cTnI and myo levels [80]; and cardiopulmonary resuscitation can lead to elevated markers of myocardial damage [18, 81–83]).

Another important factor is putrefaction, including blood degradation. Thus, in the present study, postmortem changes (i.e., macroscopic blood quality, putrefaction grade, PMI) were analyzed to define criteria for the selection of blood samples suitable for postmortem cBM POCT. The results indicate that a PMI ≤ 96 h seems acceptable if the blood is free of clots and fat globules obtained from a body with putrefaction not exceeding extensive discolorations of the skin and resolved rigor mortis. When these criteria were applied, the present study revealed an error rate of only approximately 22.9%, although in legal medicine casework, the exact PMI is often not known and must be estimated. Thus, the presented criteria may help to save resources and reduce measurement errors due to insufficient sample quality. However, while these results are interpreted, the PMI duration must be considered, as it can severely influence biomarker levels [84].

The stability of different biomarkers must also be considered in postmortem cBM POCT. In the present study, information on some cBM levels shortly before death was available for 13 patients. Generally, when the ante- and postmortem biomarker levels were compared, an average decrease of approximately 15% was observed. This finding suggests that postmortem tissue degradation does not lead to false-positive POCT results. In contrast, the findings suggest that the high levels above known normal limits observed postmortem are likely to have already existed before death. However, the ante- and postmortem comparison of cTnI levels showed that a PMI ≥ 36 h was usually associated with increased cTnI levels. Taken together, these findings suggest that postmortem tissue degradation affects markers of myocardial damage more than markers of wall tension.

Although a longer half-life of NT-proBNP than of BNP has been reported [24], reliable results in the analyzed postmortem blood samples were observed for both biomarkers despite a decrease to approximately 85% of the levels measured before death. This finding aligns with clinical observations that both markers are stable over time [85, 86]. For NT-proBNP, even stability during freezing and thawing cycles has been demonstrated [87].

Some previous studies revealed that postmortem blood samples are not suitable for the analysis of biomarkers such as cTnI [88] or that postmortem blood samples are not suitable for any “standard biochemical assay” at all [89]. However, the present study revealed that EDTA-anticoagulated and postmortem blood selected on the basis of predefined criteria can be suitable for cBM POCT.

Prior studies revealed the high importance of the actual sampling site for postmortem cBM analysis (Table 1). For example, a comparative analysis of blood samples obtained from the left heart, right heart, peripheral veins, and pericardial fluid revealed higher levels of myocardial injury markers in samples obtained from or adjacent to the heart than in those obtained from peripheral venous blood [16]. Thus, the blood samples obtained from the intrapericardial inferior vena cava seem likely to be influenced by myocardial decomposition. This sampling method, however, is associated with a high likelihood of actually obtaining a sample compared with sampling from other sites (e.g., femoral veins). Here again, this finding is fundamental for the analysis of consecutive cases and thus for defining selection criteria, such as those used in the present study.

Taken together, our findings indicate that postmortem cBM levels must be interpreted contextually, and all available information, including the sampling site, properties of the analyzed marker, autopsy findings, investigative results, etc., must be considered by legal medicine experts.

### Preanalytical phase in postmortem cBM POCT

As in previous studies [16], the present study revealed changes in cBM levels 24 h later (T_2a_ and T_2b_) than initial measurement (T_1a_ and T_1b_; mean changes within CI). Interestingly, the different half-lives of the cardiac biomarkers (half-life for each biomarker according to the literature: BNP = 20 min [45], NT-proBNP = 120 min [45], cTnI = 360 min [90], Myo = 120 to 180 min [91], CK-MB = 660 min [92]) did not seem to influence postmortem stability for POCT within 24 h after autopsy. The comparable results of double measurements at different time points (i.e., T_1a_ vs. T_1b_ and T_2a_ vs. T_2b_) indicate reliable postmortem POCT results using blood samples selected on the basis of predefined criteria.

### Postmortem assessment of markers of myocardial damage

Increased myo blood levels can be found in muscle cell tears (e.g., trauma [43]) in general. Thus, autolysis and noncardiac muscle damage are likely to evoke false-positive test results. Therefore, lower myo levels seem more indicative and of higher diagnostic value, for example, to exclude myocardial ischemic damage in the postmortem setting. Accordingly, higher NPVs were found in cases of cardiac death (81.1%) in the present analysis. Additionally, CK-MB blood levels are frequently elevated in noncardiac diseases such as skeletal muscle trauma [93], convulsive seizures [94], and exercise [95]. Accordingly, the present study revealed higher CK-MB levels in many patients without autoptic evidence of primary cardiac death, and the specificity (68.35%) and PPV (58.3%) were rather low. As the present study revealed stable CK-MB levels, low values are likewise of high diagnostic value (NPV = 87.1%). Although cTn levels are typically used in the context of myocardial ischemia [41], they indicate cardiomyocyte tearing in general. Correspondingly, postmortem studies revealed elevated cTn levels [96], likely due to autolysis. Thus, low cTnI blood levels (i.e., negative POCT results) can help to rule out cardiac death associated with myocardial damage.

### Postmortem assessment of markers of increased wall tension

Clinically, NT-proBNP and BNP are used for HF diagnosis and monitoring [51], with equal diagnostic performance of both biomarkers [45]. In the present postmortem analysis, however, NT-proBNP was superior to BNP in terms of the identification of cardiac death in general (Table 5). This finding might be explained by accompanying conditions that change the NT-proBNP/BNP ratio, such as age, impaired renal function, atrial fibrillation [61] or the longer half-life of NT-pro BNP (120 min; BNP = 20 min [45]). In the classification of cardiac death associated with congestive HF, both BNP and NT-proBNP showed comparable diagnostic performance (Table 4).

In addition to congestion, myocardial ischemia influences the natriuretic peptide system [97]. For example, experimental induction of acute myocardial infarction rapidly led to increased BNP mRNA expression [98, 99], resulting in elevated BNP plasma levels [99]. Thus, ischemia is a potential confounder of the present study. To determine the actual effect of hypoxia on postmortem NT-proBNP and BNP levels, studies in which deaths related solely to acute myocardial ischemia not associated with chronic coronary disease (e.g., drowning, cyanide, or carbon monoxide intoxication), deaths with evidence of chronic ischemia, and cases with signs of congestion not caused by myocardial ischemia are compared are needed.

### Postmortem diagnosis of HF

Postmortem HF is a challenging diagnosis. For example, thus far, postmortem investigations have been focused on congestive HF (for example, the present study or Sabatasso et al. 2011 [52]) but not yet on HF with preserved ejection fraction [51]. Nevertheless, autopsy is the widely accepted “gold standard” for determining the cause of death [40]. Therefore, it was chosen as the baseline for the assessment of the diagnostic performance of postmortem cBM POCT. However, postmortem cBM levels can provide clues regarding the functional state of the circulatory system around the time of death (e.g., NT-proBNP and BNP) and thus help to broaden the scope of legal medicine for organ (mal-)function diagnosis.

### Future perspectives

In addition to the assessed biomarker panel, other markers, such as atrial natriuretic peptide (ANP), have already been analyzed in clinical [100] and medico-legal research [101]. In legal medicine casework, ANP seems to be a promising candidate parameter to further improve the sometimes challenging diagnosis of drowning [70], especially in combination with BNP [19] and other noncardiac markers [102]. Thus, further research on postmortem cBM analysis will support not only the diagnosis of cardiac death itself but also other diagnoses of medico-legal relevance, such as drowning.

## Conclusion

Postmortem cBM POCT of myo, cTnI, NT-proBNP, BNP, and CK-MB levels in blood samples selected according to predefined criteria and obtained from the intrapericardial inferior vena cava provides reproducible and stable results. Blood seems suitable for postmortem cBM POCT; if the PMI is ≤ 96 h, there are no fat globules or clots in the sample, and putrefaction is limited to a maximum of extensive discolorations of the skin, decomposition fluid draining from at least one orifice, and rigor mortis being resolved. The assessed cBMs exhibited acceptable diagnostic performance. Normal levels of the assessed cBMs can be seen as indicators of a low likelihood of cardiac death in general and/or death associated with congestive HF. Elevated NT-pro-BNP and BNP levels are plausible indicators of cardiac death associated with congestive HF. Elevated levels of cTnI, Myo, and CK-MB have limited diagnostic value, whereas normal levels indicate a low likelihood of severe myocardial damage. The cBM POCT results must be interpreted in conjunction with all available information, i.e., autopsy findings, sampling site, properties of the assessed marker, medical history, other investigatory results, and other test results.

## Supplementary Information

Below is the link to the electronic supplementary material.

ESM 1 (PDF 288 KB)

## Data Availability

Anonymized data can be requested from the senior author (frank.ramsthaler@uks.eu) who performed the statistical analysis.

## Declarations

### Artificial intelligence statement

The *Grammarly* application (https://app.grammarly.com/; free version) using AI was used in the preparation of the manuscript to assist with correct phrasing and wording. No generative AI has been used.
