# Supplementary material for: Assessment of cardiac biomarker “point-of-care” testing as postmortem diagnostic tool
Source: Int J Legal Med. 2025 May 29;139(5):2577–91. doi: 10.1007/s00414-025-03517-y (PMC12354503; doi:10.1007/s00414-025-03517-y)
Supplement: Supplementary file 1 — (PDF 288 KB) [file 414_2025_3517_MOESM1_ESM.pdf]

Supplemental File

Assessment of cardiac biomarker "point-of-care" testing as post-mortem diagnostic tool

Jan Michael Federspiel[1,*], Mattias Kettner[2,3], Stefan Potente[1], Sara Heinbuch[4], Constantin Lux[2], Marcel A. Verhoff[2], Frank Ramsthaler[1]

1. Institute for Legal Medicine, Saarland University, Faculty of Medicine, Campus Homburg, Building 49.1, Kirrberger Straße, 66421 Homburg, Saarland, Germany.
2. Institute of Legal Medicine, University Hospital of Frankfurt, Goethe University, Kennedyallee 104, 60596 Frankfurt (Main), Hessen, Germany.
3. Department of Forensic Medicine and Imaging, Institute of Forensic Medicine, University of Zurich, Winterthurerstrasse 190/52, 8057, Zurich, Switzerland.
4. Department of Psychiatry, Clinic for Psychiatry, Psychotherapy and Psychosomatics, SHG-Kliniken Sonnenberg, Saarbrücken, Germany.

* **Corresponding author:** Jan Michael Federspiel, MD. Institute of Legal Medicine, Saarland University, Medical Faculty, Campus Homburg, Building 49.1, E-mail: jmfederspiel@outlook.com, Phone (secretary): 0049 6841 16 26300; Fax (secretary): 0049 6841 16 26314.

Appendix A – Abbreviations list

AUC	area under the curve
BNP	brain-type natriuretic peptide
cBM	cardiac biomarker
CI	95%-confidence interval
CK-MB	creatine kinase muscle-brain-type
CV	coefficient of variation
EDTA	ethylenediaminetetraacetic acid
(hs) cTn(I/T)	(high sensitivity) cardiac Troponin (T/I)
HF	heart failure
LH(+/-)	(positive/negative) likelihood ratio
LoA	limit of agreement
Myo	myoglobin
NPV	negative predictive value
NT-proBNP	N-terminal pro-brain-type natriuretic peptide
PMI	postmortem interval
POCT	point-of-care-testing
PPV	positive predictive value
ROC	receiver operating characteristics
SD	standard deviation
SOB	shortness of breath.

Appendix B – Systematic literature search

Database: Pubmed

Last database query: 5th March 2025 09:55am

==

Search algorithm 1: (postmortem) AND (cardiac biomarkers) AND (quality)

Identification of 14 publications (txt output of PubMed):

1: Ding Z, Wei Q, Liu C, Zhang H, Huang F. The Quality Changes and Proteomic Analysis of Cattle Muscle Postmortem during Rigor Mortis. Foods. 2022 Jan 13;11(2):217. doi: 10.3390/foods11020217. PMID: 35053949; PMCID: PMC8775072.

2: Xu C, Zhang T, Zhu B, Cao Z. Diagnostic role of postmortem CK-MB in cardiac death: a systematic review and meta-analysis. Forensic Sci Med Pathol. 2020 Jun;16(2):287-294. doi: 10.1007/s12024-020-00232-5. Epub 2020 Mar 19. PMID: 32193705.

3: Cao Z, Zhao M, Xu C, Zhang T, Jia Y, Wang T, Zhu B. Evaluation of Agonal Cardiac Function for Sudden Cardiac Death in Forensic Medicine with Postmortem Brain Natriuretic Peptide (BNP) and NT-proBNP: A Meta-analysis. J Forensic Sci. 2020 May;65(3):686-691. doi: 10.1111/1556-4029.14232. Epub 2019 Nov 18. PMID: 31738462.

4: Gross AL, Gray-Edwards HL, Bebout CN, Ta NL, Nielsen K, Brunson BL, Lopez Mercado KR, Osterhoudt DE, Batista AR, Maitland S, Seyfried TN, Sena-Esteves M, Martin DR. Intravenous delivery of adeno-associated viral gene therapy in feline GM1 gangliosidosis. Brain. 2022 Apr 18;145(2):655-669. doi: 10.1093/brain/awab309. PMID: 34410345.

5: Montanari E, Giorgetti R, Busardò FP, Giorgetti A, Tagliabracci A, Alessandrini F. Suitability of miRNA assessment in postmortem interval estimation. Eur Rev Med Pharmacol Sci. 2021 Feb;25(4):1774-1787. doi: 10.26355/eurrev_202102_25069. PMID: 33660786.

6: Kami D, Kitani T, Nakata M, Gojo S. Cardiac mesenchymal progenitors from postmortem cardiac tissues retained cellular characterization. Transplant Proc. 2014 May;46(4):1194-7. doi: 10.1016/j.transproceed.2013.11.110. PMID: 24815158.

7: Jangaard N, Sarkisian L, Saaby L, Mikkelsen S, Lassen AM, Marcussen N, Thomsen JL, Diederichsen ACP, Thygesen K, Mickley H. Incidence, Frequency, and Clinical Characteristics of Type 3 Myocardial Infarction in Clinical Practice. Am J Med. 2017 Jul;130(7):862.e9-862.e14. doi: 10.1016/j.amjmed.2016.12.034. Epub 2017 Feb 1. PMID: 28159605.

8: Zwirner J, Anders S, Bohnert S, Burkhardt R, Da Broi U, Hammer N, Pohlers D, Tse R, Ondruschka B. Screening for Fatal Traumatic Brain Injuries in Cerebrospinal Fluid Using Blood-Validated CK and CK-MB Immunoassays. Biomolecules. 2021 Jul 20;11(7):1061. doi: 10.3390/biom11071061. PMID: 34356685; PMCID: PMC8301791.

9: Woydt L, Bernhard M, Kirsten H, Burkhardt R, Hammer N, Gries A, Dreßler J, Ondruschka B. Intra-individual alterations of serum markers routinely used in forensic pathology depending on increasing postmortem interval. Sci Rep. 2018 Aug 24;8(1):12811. doi: 10.1038/s41598-018-31252-5. PMID: 30143737; PMCID: PMC6109050.

10: Mora-Ortiz M, Trichard M, Oregioni A, Claus SP. Thanatometabolomics: introducing NMR-based metabolomics to identify metabolic biomarkers of the time of death. Metabolomics. 2019 Mar 5;15(3):37. doi: 10.1007/s11306-019-1498-1. PMID: 30834988; PMCID: PMC6476858.

11: Rużycka M, Giebułtowicz J, Fudalej M, Krajewski P, Wroczyński P. Application of 2-Aminothiazoline-4-carboxylic Acid as a Forensic Marker of Cyanide Exposure. Chem Res Toxicol. 2017 Feb 20;30(2):516-523. doi: 10.1021/acs.chemrestox.6b00219. Epub 2017 Feb 7. PMID: 28080046.

12: Noutsias M, Pauschinger M, Ostermann K, Escher F, Blohm JH, Schultheiss H, Kühl U. Digital image analysis system for the quantification of infiltrates and cell adhesion molecules in inflammatory cardiomyopathy. Med Sci Monit. 2002 May;8(5):MT59-71. PMID: 12011783.

13: Casas E, Keele JW, Shackelford SD, Koohmaraie M, Sonstegard TS, Smith TP, Kappes SM, Stone RT. Association of the muscle hypertrophy locus with carcass traits in beef cattle. J Anim Sci. 1998 Feb;76(2):468-73. doi: 10.2527/1998.762468x. PMID: 9498354.

14: Hoffman JI, Thorne MA, Trathan PN, Forcada J. Transcriptome of the dead: characterisation of immune genes and marker development from necropsy samples in a free-ranging marine mammal. BMC Genomics. 2013 Jan 24;14:52. doi: 10.1186/1471-2164-14-52. PMID: 23347513; PMCID: PMC3563519.

Summary: No study is available covering quality control and establishment of the postmortem cardiac biomarker analysis.

==

Search algorithm 2: (postmortem) AND (cardiac biomarkers) AND (establish)

Identification of 86 publications (txt output of PubMed):

1: Charidimou A, Boulouis G, Frosch MP, Baron JC, Pasi M, Albucher JF, Banerjee G, Barbato C, Bonneville F, Brandner S, Calviere L, Caparros F, Casolla B, Cordonnier C, Delisle MB, Deramecourt V, Dichgans M, Gokcal E, Herms J, Hernandez-Guillamon M, Jäger HR, Jaunmuktane Z, Linn J, Martinez-Ramirez S, Martínez-Sáez E, Mawrin C, Montaner J, Moulin S, Olivot JM, Piazza F, Puy L, Raposo N, Rodrigues MA, Roeber S, Romero JR, Samarasekera N, Schneider JA, Schreiber S, Schreiber F, Schwall C, Smith C, Szalardy L, Varlet P, Viguier A, Wardlaw JM, Warren A, Wollenweber FA, Zedde M, van Buchem MA, Gurol ME, Viswanathan A, Al-Shahi Salman R, Smith EE, Werring DJ, Greenberg SM. The Boston criteria version 2.0 for cerebral amyloid angiopathy: a multicentre, retrospective, MRI-neuropathology diagnostic accuracy study. Lancet Neurol. 2022 Aug;21(8):714-725. doi: 10.1016/S1474-4422(22)00208-3. PMID: 35841910; PMCID: PMC9389452.

2: Barberi C, van den Hondel KE. The use of cardiac troponin T (cTnT) in the postmortem diagnosis of acute myocardial infarction and sudden cardiac death: A systematic review. Forensic Sci Int. 2018 Nov;292:27-38. doi: 10.1016/j.forsciint.2018.09.002. Epub 2018 Sep 17. PMID: 30269044.

3: Nusair SD, Joukhan AN, Rashaid AB, Rababa'h AM. Methomyl induced effect on fortilin and S100A1 in serum and cardiac tissue: Potential biomarkers of toxicity. Hum Exp Toxicol. 2019 Mar;38(3):371-377. doi: 10.1177/0960327118814153. Epub 2018 Nov 25. PMID: 30472887.

4: Huang Y, Qiu H, Chen W, Meng Z, Cai Y, Qiao D, Yue X. Identification of TRAF2, CAMK2G, and TIMM17A as biomarkers distinguishing mechanical asphyxia from sudden cardiac death base on 4D-DIA Proteomics: A pilot study. J Pharm Biomed Anal. 2025 Feb 3;258:116730. doi: 10.1016/j.jpba.2025.116730. Epub ahead of

print. PMID: 39921950.

5: Bañón R, Hernández-Romero D, Navarro E, Pérez-Cárceles MD, Noguera-Velasco JA, Osuna E. Combined determination of B-type natriuretic peptide and high-sensitivity troponin I in the postmortem diagnosis of cardiac disease. ForensicSci Med Pathol. 2019 Dec;15(4):528-535. doi: 10.1007/s12024-019-00150-1. Epub 2019 Aug 30. PMID: 31471870.

6: Hernández-Romero D, Valverde-Vázquez MDR, Hernández Del Rincón JP, Noguera-Velasco JA, Pérez-Cárceles MD, Osuna E. Diagnostic Application of Postmortem Cardiac Troponin I Pericardial Fluid/Serum Ratio in Sudden Cardiac Death. Diagnostics (Basel). 2021 Mar 30;11(4):614. doi: 10.3390/diagnostics11040614. PMID: 33808170; PMCID: PMC8067238.

7: Finn AV, Nakano M, Narula J, Kolodgie FD, Virmani R. Concept of vulnerable/unstable plaque. Arterioscler Thromb Vasc Biol. 2010 Jul;30(7):1282-92. doi: 10.1161/ATVBAHA.108.179739. PMID: 20554950.

8: Moridi M, Magnusson C, Zilg B. Cardiac troponin T as a postmortem biomarker for acute myocardial infarction. Forensic Sci Int. 2022 Dec;341:111506. doi: 10.1016/j.forsciint.2022.111506. Epub 2022 Oct 28. PMID: 36368163.

9: Tsamourgelis A, Swann P, Chouliaras L, O'Brien JT. From protein biomarkers to proteomics in dementia with Lewy Bodies. Ageing Res Rev. 2023 Jan;83:101771. doi: 10.1016/j.arr.2022.101771. Epub 2022 Nov 1. PMID: 36328346.

10: Sacco MA, Gualtieri S, Grimaldi G, Monterossi MD, Aquila VR, Tarallo AP, Verrina MC, Ranno F, Gratteri S, Aquila I. The Role of Cardiac Troponins in Postmortem Diagnosis of Myocardial Ischemia: A Systematic Review. Int J Mol Sci. 2024 Dec 26;26(1):105. doi: 10.3390/ijms26010105. PMID: 39795962; PMCID: PMC11719723.

11: Durcan R, Donaghy P, Osborne C, Taylor JP, Thomas AJ. Imaging in prodromal dementia with Lewy bodies: Where do we stand? Int J Geriatr Psychiatry. 2019 May;34(5):635-646. doi: 10.1002/gps.5071. Epub 2019 Mar 15. PMID: 30714199.

12: Kobayashi R, Iwata-Endo K, Fujishiro H. Clinical presentations and diagnostic application of proposed biomarkers in psychiatric-onset prodromal dementia with Lewy bodies. Psychogeriatrics. 2024 Jul;24(4):1004-1022. doi: 10.1111/psyg.13147. Epub 2024 Jun 4. PMID: 38837629.

13: Cut TG, Ciocan V, Novacescu D, Voicu A, Marinescu AR, Lazureanu VE, Muresan CO, Enache A, Dumache R. Autopsy Findings and Inflammatory Markers in SARS-CoV-2: A Single-Center Experience. Int J Gen Med. 2022 Dec 28;15:8743-8753. doi: 10.2147/IJGM.S389300. PMID: 36597439; PMCID: PMC9805743.

14: Tu C, Du T, Ye X, Shao C, Xie J, Shen Y. Using miRNAs and circRNAs to estimate PMI in advanced stage. Leg Med (Tokyo). 2019 May;38:51-57. doi: 10.1016/j.legalmed.2019.04.002. Epub 2019 Apr 2. PMID: 30986695.

15: Takahashi M, Ikemura M, Oka T, Uchihara T, Wakabayashi K, Kakita A, Takahashi H, Yoshida M, Toru S, Kobayashi T, Orimo S. Quantitative correlation between cardiac MIBG uptake and remaining axons in the cardiac sympathetic nerve in Lewy body disease. J Neurol Neurosurg Psychiatry. 2015 Sep;86(9):939-44. doi: 10.1136/jnnp-2015-310686. Epub 2015 May 2. PMID: 25935891.

16: Cousins KAQ, Shaw LM, Chen-Plotkin A, Wolk DA, Van Deerlin VM, Lee EB, McMillan CT, Grossman M, Irwin DJ. Distinguishing Frontotemporal Lobar Degeneration Tau From TDP-43 Using Plasma Biomarkers. JAMA Neurol. 2022 Nov 1;79(11):1155-1164. doi: 10.1001/jamaneurol.2022.3265. PMID: 36215050; PMCID: PMC9552044.

17: Martínez-Jiménez D, Hernández Del Rincón JP, Sabater-Molina M, Pérez-Martínez C, Torres C, Pérez-Cárceles MD, Luna A. Postmortem study of adrenomedullin and cortisol in femoral serum and pericardial fluid related to acute pulmonary edema. Int J Legal Med. 2025 Jan;139(1):353-359. doi: 10.1007/s00414-024-03337-6. Epub 2024 Sep 26. PMID: 39325159; PMCID: PMC11732926.

18: Kim SY, Lee S, Park JT, Lee SJ, Kim HS. Postmortem-Derived Exosomal MicroRNA 486-5p as Potential Biomarkers for Ischemic Heart Disease Diagnosis. Int J Mol Sci. 2024 Sep 5;25(17):9619. doi: 10.3390/ijms25179619. PMID: 39273565; PMCID: PMC11395318.

19: Liu N, Xie L, Xiao P, Chen X, Kong W, Lou Q, Chen F, Lu X. Cardiac fibroblasts secrete exosome microRNA to suppress cardiomyocyte pyroptosis in myocardial ischemia/reperfusion injury. Mol Cell Biochem. 2022 Apr;477(4):1249-1260. doi: 10.1007/s11010-021-04343-7. Epub 2022 Feb 4. PMID: 35119583; PMCID: PMC8913441.

20: Djoussé L, Biggs ML, Ix JH, Kizer JR, Lemaitre RN, Sotoodehnia N, Zieman SJ, Mozaffarian D, Tracy RP, Mukamal KJ, Siscovick DS. Nonesterified fatty acids and risk of sudden cardiac death in older adults. Circ Arrhythm Electrophysiol. 2012 Apr;5(2):273-8. doi: 10.1161/CIRCEP.111.967661. Epub 2012 Jan 26. PMID: 22281952; PMCID: PMC3329563.

21: Buja LM, Zehr B, Lelenwa L, Ogechukwu E, Zhao B, Dasgupta A, Barth RF. Clinicopathological complexity in the application of the universal definition of myocardial infarction. Cardiovasc Pathol. 2020 Jan-Feb;44:107153. doi: 10.1016/j.carpath.2019.107153. Epub 2019 Oct 10. PMID: 31760238.

22: Peng D, Lv M, Li Z, Tian H, Qu S, Jin B, Long B, Liang W, Zhang L. Postmortem interval determination using mRNA markers and DNA normalization. Int J Legal Med. 2020 Jan;134(1):149-157. doi: 10.1007/s00414-019-02199-7. Epub 2019 Nov 26. PMID: 31773316.

23: Lv YH, Ma JL, Pan H, Zeng Y, Tao L, Zhang H, Li WC, Ma KJ, Chen L. Estimation of the human postmortem interval using an established rat mathematical model and multi-RNA markers. Forensic Sci Med Pathol. 2017 Mar;13(1):20-27. doi: 10.1007/s12024-016-9827-4. Epub 2016 Dec 28. PMID: 28032211.

24: Tao L, Ma J, Han L, Xu H, Zeng Y, Yehui L, Li W, Ma K, Xiao B, Chen L. Early postmortem interval estimation based on Cdc25b mRNA in rat cardiac tissue. Leg Med (Tokyo). 2018 Nov;35:18-24. doi: 10.1016/j.legalmed.2018.09.004. Epub 2018 Sep 10. PMID: 30237007.

25: Javan GT, Finley SJ, Moretti M, Visonà SD, Mezzari MP, Green RL. COVID-19 and brain-heart-lung microbial fingerprints in Italian cadavers. Front Mol Biosci. 2023 Jun 14;10:1196328. doi: 10.3389/fmolb.2023.1196328. PMID: 37388248; PMCID: PMC10300556.

26: Graham-Brown MP, Patel AS, Stensel DJ, March DS, Marsh AM, McAdam J, McCann GP, Burton JO. Imaging of Myocardial Fibrosis in Patients with End-Stage Renal Disease: Current Limitations and Future Possibilities. Biomed Res Int. 2017;2017:5453606. doi: 10.1155/2017/5453606. Epub 2017 Mar 2. PMID: 28349062; PMCID: PMC5352874.

27: Kishore S, Gupta SK, Arava SK, Mridha AR, Jaiswal AK, Sikary AK, Bharti DR, Behera C. Biochemical findings in sudden unexpected death in epilepsy: Hospital based case-control study. J Forensic Leg Med. 2020 Jan;69:101884. doi: 10.1016/j.jflm.2019.101884. Epub 2019 Nov 9. PMID: 31739178.

28: Firl CEM, Halushka M, Fraser N, Masson M, Cuneo BF, Saxena A, Clancy R, Buyon J. Contribution of S100A4-expressing fibroblasts to anti-SSA/Ro-associated atrioventricular nodal calcification and soluble S100A4 as a biomarker of clinical severity. Front Immunol. 2023 Apr 6;14:1114808. doi: 10.3389/fimmu.2023.1114808. PMID: 37090702; PMCID: PMC10117984.

29: Batalis NI, Marcus BJ, Papadea CN, Collins KA. The role of postmortem cardiac markers in the diagnosis of acute myocardial infarction. J Forensic Sci. 2010 Jul;55(4):1088-91. doi: 10.1111/j.1556-4029.2010.01368.x. Epub 2010 Mar 25. PMID: 20345772.

30: Aljakna A, Fracasso T, Sabatasso S. Molecular tissue changes in early myocardial ischemia: from pathophysiology to the identification of new diagnostic markers. Int J Legal Med. 2018 Mar;132(2):425-438. doi: 10.1007/s00414-017-1750-z. Epub 2018 Jan 23. PMID: 29362873.

31: Berber R, Abdel-Gadir A, Rosmini S, Captur G, Nordin S, Culotta V, Palla L, Kellman P, Lloyd GW, Skinner JA, Moon JC, Manisty C, Hart AJ. Assessing for Cardiotoxicity from Metal-on-Metal Hip Implants with Advanced Multimodality Imaging Techniques. J Bone Joint Surg Am. 2017 Nov 1;99(21):1827-1835. doi: 10.2106/JBJS.16.00743. PMID: 29088037; PMCID: PMC6948834.

32: Liu H, Wang Q, Zhao Z, Xie Y, Ding S, Wang Z. The Clinical and Medicolegal Analysis of Electrical Shocked Rats: Based on the Serological and Histological Methods. Biomed Res Int. 2016;2016:4896319. doi: 10.1155/2016/4896319. Epub 2016 Aug 25. PMID: 27648446; PMCID: PMC5014935.

33: Palmiere C, Egger C. Usefulness of pericardial and pleural fluids for the postmortem diagnosis of sepsis. J Forensic Leg Med. 2014 Nov;28:15-8. doi: 10.1016/j.jflm.2014.09.006. Epub 2014 Sep 19. PMID: 25440141.

34: Popescu LM, Manole CG, Gherghiceanu M, Ardelean A, Nicolescu MI, Hinescu ME, Kostin S. Telocytes in human epicardium. J Cell Mol Med. 2010 Aug;14(8):2085-93. doi: 10.1111/j.1582-4934.2010.01129.x. Epub 2010 Jul 13. PMID: 20629996; PMCID: PMC3823000.

35: Han D, Wang Y, Wang Y, Dai X, Zhou T, Chen J, Tao B, Zhang J, Cao F. The Tumor-Suppressive Human Circular RNA CircITCH Sponges miR-330-5p to Ameliorate Doxorubicin-Induced Cardiotoxicity Through Upregulating SIRT6, Survivin, and SERCA2a. Circ Res. 2020 Jul 31;127(4):e108-e125. doi: 10.1161/CIRCRESAHA.119.316061. Epub 2020 May 11. PMID: 32392088.

36: Palmiere C, Tettamanti C, Augsburger M, Burkhardt S, Sabatasso S, Lardi C, Werner D. Postmortem biochemistry in suspected starvation-induced ketoacidosis. J Forensic Leg Med. 2016 Aug;42:51-5. doi: 10.1016/j.jflm.2016.04.013. Epub 2016 May 4. PMID: 27239954.

37: de la Grandmaison GL. Is there progress in the autopsy diagnosis of sudden unexpected death in adults? Forensic Sci Int. 2006 Jan 27;156(2-3):138-44. doi: 10.1016/j.forsciint.2004.12.024. PMID: 16410164.

38: Ivanova AA, Gurazheva AA, Melnikova ES, Nesterets AM, Malyutina SK, Rodina IA, Maksimov VN. Verification of Single Nucleotide Polymorphisms rs34554140, rs6670279, and rs6874185 as Novel Molecular Genetic Markers of Sudden Cardiac Death. Sovrem Tekhnologii Med. 2021;13(2):40-44. doi: 10.17691/stm2021.13.2.04. Epub 2021 Jan 1. PMID: 34513075; PMCID: PMC8353714.

39: Verrier RL, Nearing BD, Olin B, Boon P, Schachter SC. Baseline elevation and reduction in cardiac electrical instability assessed by quantitative T-wave alternans in patients with drug-resistant epilepsy treated with vagus nerve stimulation in the AspireSR E-36 trial. Epilepsy Behav. 2016 Sep;62:85-9. doi: 10.1016/j.yebeh.2016.06.016. Epub 2016 Jul 21. PMID: 27450311.

40: Skinner MP. Thrombosis and thrombolysis: platelet membrane glycoproteins. Heart Lung Circ. 2007 Jun;16(3):176-9. doi: 10.1016/j.hlc.2007.03.004. Epub 2007 Apr 18. PMID: 17446123.

41: Dams-O'Connor K, Awwad HO, Hoffman S, Pugh MJ, Johnson VE, Keene CD, McGavern L, Mukherjee P, Opanashuk L, Umoh N, Sopko G, Zetterberg H. Alzheimer's Disease-Related Dementias Summit 2022: National Research Priorities for the Investigation of Post-Traumatic Brain Injury Alzheimer's Disease and Related Dementias. J Neurotrauma. 2023 Aug;40(15-16):1512-1523. doi: 10.1089/neu.2022.0514. Epub 2023 Apr 25. PMID: 36927167; PMCID: PMC10494902.

42: Vargas SO, Grudzien C, Tanasijevic MJ. Postmortem cardiac troponin-I levels predict intramyocardial damage at autopsy. J Thromb Thrombolysis. 2008 Oct;26(2):132-7. doi: 10.1007/s11239-007-0173-y. Epub 2007 Dec 7. PMID: 18064406.

43: Jacobs ER, Ross GR, Padilla N, Pan AY, Liegl M, Puzyrenko A, Lai S, Dai Q, Uche N, Rubenstein JC, North PE, Ibrahim EH, Sun Y, Felix JC, Rui H, Benjamin IJ. Profibrotic COVID-19 subphenotype exhibits enhanced localized ER-dependent HSP47⁺ expression in cardiac myofibroblasts in situ. J Mol Cell Cardiol. 2023 Dec;185:1-12. doi: 10.1016/j.yjmcc.2023.10.006. Epub 2023 Oct 14. PMID: 37839656; PMCID: PMC11000691.

44: Leifheit-Nestler M, Große Siemer R, Flasbart K, Richter B, Kirchhoff F, Ziegler WH, Klintschar M, Becker JU, Erbersdobler A, Aufricht C, Seeman T, Fischer DC, Faul C, Haffner D. Induction of cardiac FGF23/FGFR4 expression is associated with left ventricular hypertrophy in patients with chronic kidney disease. Nephrol Dial Transplant. 2016 Jul;31(7):1088-99. doi: 10.1093/ndt/gfv421. Epub 2015 Dec 17. PMID: 26681731; PMCID: PMC6388939.

45: Takasu S, Matsumoto S, Kanto Y, Iwadate K, Iwadate K. Relationship between N-terminal pro-brain natriuretic peptide concentration and heart-type fatty acid-binding protein in postmortem urine. Leg Med (Tokyo). 2024 Sep;70:102479. doi: 10.1016/j.legalmed.2024.102479. Epub 2024 Jun 26. PMID: 38943789.

46: Lee DC, Gevorgyan T, Graber HL, Pfeil DS, Xu Y, Mangla S, Barone FC, Libien J, Charchaflieh J, Kral JG, Ramirez SA, Simpson L, Barbour RL. Feasibility of near-infrared spectroscopic tomography for intraoperative functional cerebral monitoring: a primate study. J Thorac Cardiovasc Surg. 2014 Dec;148(6):3204-10.e1-2. doi: 10.1016/j.jtcvs.2014.07.041. PMID: 25439529.

47: Singh P, Rao P, Yadav SK, Gujar NL, Satpute RM, Bhattacharya R. Time- and temperature-dependent changes in cytochrome c oxidase activity and cyanide concentration in excised mice organs and mice cadavers. J Forensic Sci. 2015 Jan;60 Suppl 1:S162-70. doi: 10.1111/1556-4029.12614. Epub 2014 Nov 24. PMID: 25420913.

48: Zwirner J, Anders S, Bohnert S, Burkhardt R, Da Broi U, Hammer N, Pohlers D, Tse R, Ondruschka B. Screening for Fatal Traumatic Brain Injuries in Cerebrospinal Fluid Using Blood-Validated CK and CK-MB Immunoassays. Biomolecules. 2021 Jul 20;11(7):1061. doi: 10.3390/biom11071061. PMID: 34356685; PMCID: PMC8301791.

49: Riezzo I, Ventura F, D'Errico S, Neri M, Turillazzi E, Fineschi V. Arrhythmogenesis and diagnosis of cardiac sarcoidosis. An immunohistochemical study in a sudden cardiac death. Forensic Sci Int. 2009 Jan 10;183(1-3):e1-5. doi: 10.1016/j.forsciint.2008.09.020. Epub 2008 Nov 18. PMID: 19019592.

50: Nascimento AF, Winters GL, Pinkus GS. Primary cardiac lymphoma: clinical, histologic, immunophenotypic, and genotypic features of 5 cases of a rare disorder. Am J Surg Pathol. 2007 Sep;31(9):1344-50. doi: 10.1097/PAS.0b013e3180317341. PMID: 17721189.

51: Kim BM, Park SU, Schmelzer L, Yang SB, Lee SD, Kim MY, Naue J, Lee HY. DNA methylation-based organ tissue identification: Marker identification, SNaPshot multiplex assay development, and interlaboratory comparison. Forensic Sci Int Genet. 2024 Jul;71:103052. doi: 10.1016/j.fsigen.2024.103052. Epub 2024 Apr 22. PMID: 38678764.

52: Zhu BL, Ishikawa T, Michiue T, Quan L, Maeda H. Postmortem serum endotoxin level in relation to the causes of death. Leg Med (Tokyo). 2005 Mar;7(2):103-9. doi: 10.1016/j.legalmed.2004.08.004. PMID: 15708333.

53: Muta H, Sugita Y, Ohshima K, Otsubo H. Primary malignant pericardial sarcomatoid mesothelioma: An autopsy report. Pathol Int. 2017 Jun;67(6):311-315. doi: 10.1111/pin.12535. Epub 2017 May 1. PMID: 28463437.

54: Kostopoulos CG, Spiroglou SG, Varakis JN, Apostolakis E, Papadaki HH. Adiponectin/T-cadherin and apelin/APJ expression in human arteries and periadventitial fat: implication of local adipokine signaling in atherosclerosis? Cardiovasc Pathol. 2014 May-Jun;23(3):131-8. doi: 10.1016/j.carpath.2014.02.003. Epub 2014 Feb 25. PMID: 24675084.

55: Pelsers MM, Hanhoff T, Van der Voort D, Arts B, Peters M, Ponds R, Honig A, Rudzinski W, Spener F, de Kruijk JR, Twijnstra A, Hermens WT, Menheere PP, Glatz JF. Brain- and heart-type fatty acid-binding proteins in the brain: tissue distribution and clinical utility. Clin Chem. 2004 Sep;50(9):1568-75. doi: 10.1373/clinchem.2003.030361. Epub 2004 Jun 24. PMID: 15217991.

56: Cronberg T, Rundgren M, Westhall E, Englund E, Siemund R, Rosén I, Widner H, Friberg H. Neuron-specific enolase correlates with other prognostic markers after cardiac arrest. Neurology. 2011 Aug 16;77(7):623-30. doi: 10.1212/WNL.0b013e31822a276d. Epub 2011 Jul 20. PMID: 21775743.

57: Joshi MS, Tong L, Cook AC, Schanbacher BL, Huang H, Han B, Ayers LW, Bauer JA. Increased myocardial prevalence of C-reactive protein in human coronary heart disease: direct effects on microvessel density and endothelial cell survival. Cardiovasc Pathol. 2012 Sep-Oct;21(5):428-35. doi: 10.1016/j.carpath.2011.12.003. Epub 2012 Jan 28. PMID: 22285194; PMCID: PMC3899797.

58: Grasmeyer S, Madea B. Immunohistochemical diagnosis of myocarditis on (infantile) autopsy material: Does it improve the diagnosis? Forensic Sci Med Pathol. 2015 Jun;11(2):168-76. doi: 10.1007/s12024-015-9675-7. Epub 2015 Apr 17. PMID: 25894115.

59: Tanaka N. [Induction mechanism of shock: applying the etiology in judgment of the cause of death in forensic practice]. Nihon Hoigaku Zasshi. 2004 Sep;58(2):130-40. Japanese. PMID: 15526767.

60: Guo Q, Qi Q, You Q, Gu H, Zhao L, Wu Z. Toxicological studies of gambogic acid and its potential targets in experimental animals. Basic Clin Pharmacol Toxicol. 2006 Aug;99(2):178-84. doi: 10.1111/j.1742-7843.2006.pto_485.x. PMID: 16918721.

61: Gow DJ, Gow AG, Bell R, Spratt D, Cash R, Ricketts S, Archer J, Mellanby RJ. Serum cardiac troponin I in dogs with primary immune-mediated haemolytic anaemia. J Small Anim Pract. 2011 May;52(5):259-64. doi: 10.1111/j.1748-5827.2011.01061.x. PMID: 21539571.

62: Rużycka M, Giebułtowicz J, Fudalej M, Krajewski P, Wroczyński P. Application of 2-Aminothiazoline-4-carboxylic Acid as a Forensic Marker of Cyanide Exposure. Chem Res Toxicol. 2017 Feb 20;30(2):516-523. doi: 10.1021/acs.chemrestox.6b00219. Epub 2017 Feb 7. PMID: 28080046.

63: Li W, Chang Y, Han L, Liu X, Cai J, Zha L, Guo Y, Ding Y. Trimethylamine in postmortem tissues as a predictor of postmortem interval estimation using the GC method. Leg Med (Tokyo). 2018 Nov;35:80-85. doi: 10.1016/j.legalmed.2018.09.011. Epub 2018 Sep 22. PMID: 30292099.

64: Quan L, Zhu BL, Ishikawa T, Michiue T, Zhao D, Li DR, Ogawa M, Maeda H. Postmortem serum erythropoietin levels in establishing the cause of death and survival time at medicolegal autopsy. Int J Legal Med. 2008 Nov;122(6):481-7. doi: 10.1007/s00414-008-0276-9. Epub 2008 Aug 6. PMID: 18682967.

65: Galassi A, Turatello L, De Salvia A, Neri M, Turillazzi E, La Russa R, Viola RV, Frati P, Fineschi V. Septic cardiomyopathy: The value of lactoferrin and CD15 as specific markers to corroborate a definitive diagnosis. Int

J Immunopathol Pharmacol. 2018 Jan-Dec;32:2058738418776526. doi: 10.1177/2058738418776526. PMID: 29809052; PMCID: PMC5977426.

66: Fais P, Mazzotti MC, Teti G, Boscolo-Berto R, Pelotti S, Falconi M. HIF1α protein and mRNA expression as a new marker for post mortem interval estimation in human gingival tissue. J Anat. 2018 Jun;232(6):1031-1037. doi: 10.1111/joa.12800. Epub 2018 Mar 5. PMID: 29504141; PMCID: PMC5980163.

67: Niccoli G, Schiavino D, Belloni F, Ferrante G, La Torre G, Conte M, Cosentino N, Montone RA, Sabato V, Burzotta F, Trani C, Leone AM, Porto I, Pieroni M, Patriarca G, Crea F. Pre-intervention eosinophil cationic protein serum levels predict clinical outcomes following implantation of drug-eluting stents. Eur Heart J. 2009 Jun;30(11):1340-7. doi: 10.1093/eurheartj/ehp120. Epub 2009 Apr 21. PMID: 19383735.

68: Anastasakis A, Papatheodorou E, Ritsatos K, Protonotarios N, Rentoumi V, Gatzoulis K, Antoniades L, Agapitos E, Koutsaftis P, Spiliopoulou C, Tousoulis D. Sudden unexplained death in the young: epidemiology, aetiology and value of the clinically guided genetic screening. Europace. 2018 Mar 1;20(3):472-480. doi: 10.1093/europace/euw362. PMID: 28177452.

69: Madjid M, Vela D, Khalili-Tabrizi H, Casscells SW, Litovsky S. Systemic infections cause exaggerated local inflammation in atherosclerotic coronary arteries: clues to the triggering effect of acute infections on acute coronary syndromes. Tex Heart Inst J. 2007;34(1):11-8. PMID: 17420787; PMCID: PMC1847934.

70: Grøgaard HK, Sigurjonsson OE, Brekke M, Kløw NE, Landsverk KS, Lyberg T, Eriksen M, Egeland T, Ilebekk A. Cardiac accumulation of bone marrow mononuclear progenitor cells after intracoronary or intravenous injection in pigs subjected to acute myocardial infarction with subsequent reperfusion. Cardiovasc Revasc Med. 2007 Jan-Mar;8(1):21-7. doi: 10.1016/j.carrev.2006.09.001. PMID: 17293265.

71: Chambers SE, Geirsson RT, Stewart RJ, Wannapirak C, Muir BB. Audit of a screening service for fetal abnormalities using early ultrasound scanning and maternal serum alpha-fetoprotein estimation combined with selective detailed scanning. Ultrasound Obstet Gynecol. 1995 Mar;5(3):168-73. doi: 10.1046/j.1469-0705.1995.05030168.x. PMID: 7540492.

72: Mayer F, Pröpper S, Ritz-Timme S. Dityrosine, a protein product of oxidative stress, as a possible marker of acute myocardial infarctions. Int J Legal Med. 2014 Sep;128(5):787-94. doi: 10.1007/s00414-014-1015-z. Epub 2014 May 13. PMID: 24819153.

73: Raza-Ahmad A. Fibrinogen: a diagnostic marker for early ischemia. Biotech Histochem. 1994 Sep;69(5):268-72. doi: 10.3109/10520299409106300. PMID: 7819421.

74: Breuckmann F, Nassenstein K, Bucher C, Konietzka I, Kaiser G, Konorza T, Naber C, Skyschally A, Gres P, Heusch G, Erbel R, Barkhausen J. Systematic analysis of functional and structural changes after coronary microembolization: a cardiac magnetic resonance imaging study. JACC Cardiovasc Imaging. 2009 Feb;2(2):121-30. doi: 10.1016/j.jcmg.2008.10.011. PMID: 19356544.

75: Birkmeier S, Thiele H, Dörr R. Management des akuten ST-Strecken-Hebungsinfarktes: Update 2013 [Management of acute myocardial infarction with ST-segment elevation: Update 2013]. Herz. 2013 Dec;38(8):889-98; quiz 899. German. doi: 10.1007/s00059-013-3941-9. PMID: 24068024.

76: Sabatasso S, Pomponio C, Fracasso T. Technical note: EnVision™ FLEX improves the detectability of depletions of myoglobin and troponin T in forensic cases of myocardial ischemia/infarction. Int J Legal Med. 2017 Nov;131(6):1643-1646. doi: 10.1007/s00414-017-1575-9. Epub 2017 Mar 23. PMID: 28337600.

77: Carpenter KH, Bonham JR, Worthy E, Variend S. Vitreous humour and cerebrospinal fluid hypoxanthine concentration as a marker of pre-mortem hypoxia in SIDS. J Clin Pathol. 1993 Jul;46(7):650-3. doi: 10.1136/jcp.46.7.650. PMID: 8157754; PMCID: PMC501396.

78: Rainio J, De Paoli G, Druid H, Kauppila R, De Giorgio F, Bortolotti F, Tagliaro F. Postmortem stability and redistribution of carbohydrate-deficient transferrin (CDT). Forensic Sci Int. 2008 Jan 30;174(2-3):161-5. doi: 10.1016/j.forsciint.2007.03.020. Epub 2007 May 1. PMID: 17475426.

79: Fickelscher I, Starke H, Schulze E, Ernst G, Kosyakova N, Mkrtchyan H, MacDermont K, Sebire N, Liehr T. A further case with a small supernumerary marker chromosome (sSMC) derived from chromosome 1--evidence

for high variability in mosaicism in different tissues of sSMC carriers. Prenat Diagn. 2007 Aug;27(8):783-5. doi: 10.1002/pd.1776. PMID: 17546703.

80: Inokuchi G, Hayakawa M, Kishimoto T, Makino Y, Iwase H. A suspected case of coronary periarteritis due to IgG4-related disease as a cause of ischemic heart disease. Forensic Sci Med Pathol. 2014 Mar;10(1):103-8. doi: 10.1007/s12024-013-9516-5. Epub 2013 Dec 12. PMID: 24337948.

81: Sotlar K, Horny HP, Lebherz J, Leser HG, Bültmann B. Assoziation einer Knochenmarksmastozytose mit extrem unreifem extramedullärem Mastzellsarkom [Association of bone marrow mastocytosis with extremely immature extramedullary mast cell sarcoma]. Pathologe. 1997 May;18(3):252-6. German. doi: 10.1007/s002920050215. PMID: 9273545.

82: Katoh M, Shigematsu H. Leiomyosarcoma of the heart and its pulmonary metastasis, both with prominent osteoclast-like multinucleated giant cells expressing tartrate-resistant acid phosphatase activity. Pathol Int. 1999 Jan;49(1):74-8. doi: 10.1046/j.1440-1827.1999.00817.x. PMID: 10227728.

83: Val-Bernal JF, Martino M, Mayorga M, Garijo MF. Prichard's structures of the fossa ovalis are age-related phenomena composed of nonreplicating endothelial cells: the cardiac equivalent of cutaneous senile angioma. APMIS. 2007 Nov;115(11):1234-40. doi: 10.1111/j.1600-0643.2007.00756.x. PMID: 18092955.

84: Altaeva AZh, Zhunisov SS, Aĭdarkulov ASh, Selivokhina NV, Kidraliev RR, Darikulova BU, Zagumennikova AA. [Changes in the biochemical, chemical and toxicological characteristics of pericardial fluid in the case of fatal narcotic intoxication]. Sud Med Ekspert. 2014 Jan-Feb;57(1):34-6. Russian. PMID: 25275183.

85: Pérez-Cárceles MD, Osuna E, Vieira DN, Luna A. Usefulness of myosin in the postmortem diagnosis of myocardial damage. Int J Legal Med. 1995;108(1):14-8. doi: 10.1007/BF01845610. PMID: 7495679.

86: Nomori H, Shimosato Y, Tsuchiya R. Diffuse malignant pericardial mesothelioma. Acta Pathol Jpn. 1985 Nov;35(6):1475-81. doi: 10.1111/j.1440-1827.1985.tb01444.x. PMID: 2418626.

Summary: No study is available covering quality control and establishment of the postmortem cardiac biomarker analysis.

==

Database: Cochrane

Last database query: 5th March 2025 11:10 am

==

Search algorithm 1 (all text search for all search terms): postmortem AND cardiac biomarkers AND quality

Identification of 6 Corchrane Reviews, and 1 Cochrane Protocol. Cochrane plain txt export without keywords and abstract:

Reviews:

Record #1 of 6 ID: CD008980 AU: Bruins Slot KMH AU: Berge E TI: Factor Xa inhibitors versus vitamin K antagonists for preventing cerebral or systemic embolism in patients with atrial fibrillation SO: Cochrane Database of Systematic Reviews YR: 2018 NO: 3 PB: John Wiley & Sons, Ltd SN: 1465-1858 CC: [Stroke] DOI: 10.1002/14651858.CD008980.pub3 US: http://dx.doi.org/10.1002/14651858.CD008980.pub3

Record #2 of 6 ID: CD009068 AU: Bourke JP AU: Bueser T AU: Quinlivan R TI: Interventions for preventing and treating cardiac complications in Duchenne and Becker muscular dystrophy and X-linked dilated cardiomyopathy SO: Cochrane Database of Systematic Reviews YR: 2018 NO: 10 PB: John Wiley & Sons, Ltd SN: 1465-1858 CC: [Neuromuscular] DOI: 10.1002/14651858.CD009068.pub3 US: http://dx.doi.org/10.1002/14651858.CD009068.pub3

Record #3 of 6 ID: CD013879 AU: Pellicori P AU: Doolub G AU: Wong CM AU: Lee KS AU: Mangion K AU: Ahmad M AU: Berry C AU: Squire I AU: Lambiase PD AU: Lyon A AU: et al. TI: COVID-19 and its cardiovascular effects: a systematic review of prevalence studies SO: Cochrane Database of Systematic Reviews YR: 2021 NO: 3 PB: John Wiley & Sons, Ltd SN: 1465-1858 CC: [Heart] DOI: 10.1002/14651858.CD013879 US: http://dx.doi.org/10.1002/14651858.CD013879

Record #4 of 6 ID: CD010633 AU: McCleery J AU: Morgan S AU: Bradley KM AU: Noel-Storr AH AU: Ansorge O AU: Hyde C TI: Dopamine transporter imaging for the diagnosis of dementia with Lewy bodies SO: Cochrane Database of Systematic Reviews YR: 2015 NO: 1 PB: John Wiley & Sons, Ltd SN: 1465-1858 CC: [Dementia and Cognitive Improvement] DOI: 10.1002/14651858.CD010633.pub2 US: http://dx.doi.org/10.1002/14651858.CD010633.pub2

Record #5 of 6 ID: CD014923 AU: Pearce RK B AU: Gontsarova A AU: Richardson D AU: Methley AM AU: Watt HClare AU: Tsang K AU: Carswell C TI: Shunting for idiopathic normal pressure hydrocephalus SO: Cochrane Database of Systematic Reviews YR: 2024 NO: 8 PB: John Wiley & Sons, Ltd SN: 1465-1858 CC: [Dementia and Cognitive Improvement] DOI: 10.1002/14651858.CD014923.pub2 US: http://dx.doi.org/10.1002/14651858.CD014923.pub2

Record #6 of 6 ID: CD003134 AU: Spagnolo P AU: Del Giovane C AU: Luppi F AU: Cerri S AU: Balduzzi S AU: Walters EH AU: D'Amico R AU: Richeldi L TI: Non-steroid agents for idiopathic pulmonary fibrosis SO: Cochrane Database of Systematic Reviews YR: 2010 NO: 9 PB: John Wiley & Sons, Ltd SN: 1465-1858 CC: [Airways] DOI: 10.1002/14651858.CD003134.pub2 US: http://dx.doi.org/10.1002/14651858.CD003134.pub2

Summary: No study is available covering quality control and establishment of the postmortem cardiac biomarker analysis.

Protocols:

Record #1 of 1 ID: CD010142 AU: Magalhães PVS AU: Dean O AU: Andreazza AC AU: Berk M AU: Kapczinski F TI: Adjunctive antioxidants for bipolar disorder SO: Cochrane Database of Systematic Reviews YR: 2012 NO: 10 PB: John Wiley & Sons, Ltd SN: 1465-1858 CC: [Common Mental Disorders] DOI: 10.1002/14651858.CD010142 US: http://dx.doi.org/10.1002/14651858.CD010142

Summary: No study is available covering quality control and establishment of the postmortem cardiac biomarker analysis.

==

Search algorithm 2 (all text search for all search terms): postmortem AND cardiac biomarkers AND establish

Identification of 4 Cochrane Reviews, and 1 Trial.

Cochrane plain txt export without keywords and abstract:

Reviews:

Record #1 of 4 ID: CD009068 AU: Bourke JP AU: Bueser T AU: Quinlivan R TI: Interventions for preventing and treating cardiac complications in Duchenne and Becker muscular dystrophy and X-linked dilated cardiomyopathy SO: Cochrane Database of Systematic Reviews YR: 2018 NO: 10 PB: John Wiley & Sons, Ltd SN: 1465-1858 CC: [Neuromuscular] DOI: 10.1002/14651858.CD009068.pub3 US: http://dx.doi.org/10.1002/14651858.CD009068.pub3

Record #2 of 4 ID: CD010633 AU: McCleery J AU: Morgan S AU: Bradley KM AU: Noel-Storr AH AU: Ansorge O AU: Hyde C TI: Dopamine transporter imaging for the diagnosis of dementia with Lewy bodies SO: Cochrane Database of Systematic Reviews YR: 2015 NO: 1 PB: John Wiley & Sons, Ltd SN: 1465-1858 CC: [Dementia and Cognitive Improvement] DOI: 10.1002/14651858.CD010633.pub2 US: http://dx.doi.org/10.1002/14651858.CD010633.pub2

Record #3 of 4 ID: CD014923 AU: Pearce RK B AU: Gontsarova A AU: Richardson D AU: Methley AM AU: Watt HClare AU: Tsang K AU: Carswell C TI: Shunting for idiopathic normal pressure hydrocephalus SO: Cochrane Database of Systematic Reviews YR: 2024 NO: 8 PB: John Wiley & Sons, Ltd SN: 1465-1858 CC: [Dementia and Cognitive Improvement] DOI: 10.1002/14651858.CD014923.pub2 US: http://dx.doi.org/10.1002/14651858.CD014923.pub2

Record #4 of 4 ID: CD003134 AU: Spagnolo P AU: Del Giovane C AU: Luppi F AU: Cerri S AU: Balduzzi S AU: Walters EH AU: D'Amico R AU: Richeldi L TI: Non-steroid agents for idiopathic pulmonary fibrosis SO: Cochrane Database of Systematic Reviews YR: 2010 NO: 9 PB: John Wiley & Sons, Ltd SN: 1465-1858 CC: [Airways] DOI: 10.1002/14651858.CD003134.pub2 US: http://dx.doi.org/10.1002/14651858.CD003134.pub2

Summary: No study is available covering quality control and establishment of the postmortem cardiac biomarker analysis.

Trial:

Record #1 of 1 ID: CN-02001062 AU: NCT04128891 TI: Study of Sacubitril/ValsarTan on MyocardIal OxygenatioN and Fibrosis in Heart Failure With Preserved Ejection Fraction SO: https://clinicaltrials.gov/show/NCT04128891 YR: 2019 XR: CTgov NCT04128891 PT: Trial registry record US: https://www.cochranelibrary.com/central/doi/10.1002/central/CN-02001062/full

Summary: No study is available covering quality control and establishment of the postmortem cardiac biomarker analysis.

==
